# A dynamical behavior of the coupled Broer-Kaup-Kupershmidt equation using two efficient analytical techniques

**DOI:** 10.1371/journal.pone.0296640

**Published:** 2024-01-31

**Authors:** Rimsha Ansar, Muhammad Abbas, Homan Emadifar, Tahir Nazir, Ahmed S. M. Alzaidi

**Affiliations:** 1 Department of Mathematics, University of Sargodha, Sargodha, Pakistan; 2 Department of Mathematics, Saveetha School of Engineering, Saveetha Institute of Medical and Technical Sciences, Chennai, Tamil Nadu, India; 3 MEU Research Unit, Middle East University, Amman, Jordan; 4 Department of Mathematics, Hamedan Branch, Islamic Azad University, Hamedan, Iran; 5 Department of Mathematics and Statistics, College of Science, Taif University, Taif, Saudi Arabia; Institute of Space Technology, PAKISTAN

## Abstract

The aim of the present study is to identify multiple soliton solutions to the nonlinear coupled Broer-Kaup-Kupershmidt (BKK) system, including beta, conformable, local-fractional, and M-truncated derivatives. The coupled Broer-Kaup-Kupershmidt system is employed for modelling nonlinear wave evolution in mathematical models of fluid dynamics, plasmic, optical, dispersive, and nonlinear long-gravity waves. The travelling wave solutions to the above model are found using the Unified and generalised Bernoulli sub-ODE techniques. By modifying certain parameter values, we may create bright soliton, squeezed bell-shaped wave, expanded v-shaped soliton, W-shaped wave, singular soliton, and periodic solutions. The four distinct kinds of derivatives are compared quite effectively using 2D line graphs. Also, contour plots and 3D graphics are given by using Mathematica 10. Lastly, any pair of propagating wave solutions has symmetrical geometrical forms.

## 1. Introduction

Nonlinear partial differential equations (NLPDEs) [1] have gained a lot of attention. For modelling phenomena, they are useful. Differential equations (DEs) [2] are one of the best instruments for explaining a variety of natural processes. NLPDEs have a wide range of uses in engineering, chemistry, optical fibres, biology, physics, fluid dynamics, and crystallography [3]. In scholarly communities, they rank among the most appealing topics. To examine and understand the nature of solutions for NLPDEs, a number of reliable computational techniques were developed. Since nonlinearity characterises all physical incidences, mathematical models are typically the most suitable way to represent such phenomena. Partial differential equations (PDEs) [4] have been modelled in order to more fully examine and understand the nature of physical processes. Each approach has its benefits. Mathematical and physical models of NLPDEs are important in the theoretical sciences [5]. Aerospace engineering, sea science, atmospheric science, and other practical disciplines need an understanding of these NLPDEs [6].

In the past several decades, handling nonlinear phenomena has significantly benefited from the direct search for multi solutions [7] to nonlinear evolution equations (NLEEs) [8]. The validation of numerical solvers in solution stability analysis can be substantially facilitated by the availability of these analytical solutions for those NLEEs [9]. For NLEEs, many kinds of wave solutions are discovered. The snoidal wave, single wave, cnoidal wave, periodic wave, solitary wave, shock wave, and solitons wave are some of these forms [10]. Solitons are waves with a uniform shape and no internal energy dispersion. Soliton has significant value in the disciplines of electromagnetic fields and communications as a result of these characteristics. In many branches of science and engineering, soliton theory is crucial. Particularly, the majority of the NLPDEs have solitons-based exact solutions [11].

The analysis of fluid dynamics [12], plasmic, optical, dispersive, and nonlinear long gravity waves are just a few of the numerous fields that employ the nonlinear BKK equation [13]. When applying the nonlinear water model to port and coastal design, the BKK approach is extremely beneficial for civil and coastal engineers. The BKK system [14], which may also be derived from the famous Kadomtsev-Petviashvili (KP) equation by the symmetry constraint, which was used to model dispersive long gravity waves propagating in two horizontal directions in shallow water of uniform depth.

The rational, hyperbolic, trigonometric, singular, periodic, and singular solutions also properly expressed the solitary patterns of the BKK equation [15]. The coupled time-fractional BKK equation [16] was converted using numerous wave transformations for four distinct operators into an ordinary differential equation, namely the beta derivative (BD) [17], M-truncated derivatives (M-TD) [18], Local fractional derivative (L-FD) [19], and conformable derivative (CD) [20], each of which produces a non-linear algebraic equation system when the technique is used. This was done in order to look at how fractional parameters affected the equation’s soliton waves in a dynamic reaction.

For the analytical solutions in this study, the (2+1)-dimensional nonlinear coupled BKK equation [13] had been used as follows:

$$\begin{array}{c} \begin{array}{c} a_{tz}-a_{yyz}+2{\left(aa_{y}\right)}_{z}+2b_{yy}=0,\\ b_{t}+b_{yy}+2{\left(ab\right)}_{y}=0. \end{array} \end{array}$$ (1)

The proposed equation in BD has the following form:

$$\begin{array}{c} \begin{array}{c} D_{\beta ,t}^{\varsigma }a_{z}-a_{yyz}+2{\left(aa_{y}\right)}_{z}+2b_{yy}=0,\\ D_{\beta ,t}^{\varsigma }b+b_{yy}+2{\left(ab\right)}_{y}=0, \end{array} \end{array}$$ (2)

where $D_{\beta ,t}^{\varsigma }$ is BD and *ς* is fractional parameter.

The proposed equation in M-TD has the following form:

$$\begin{array}{c} \begin{array}{c} D_{M,t}^{\varsigma ,\delta }a_{z}-a_{yyz}+2{\left(aa_{y}\right)}_{z}+2b_{yy}=0,\\ D_{M,t}^{\varsigma ,\delta }b+b_{yy}+2{\left(ab\right)}_{y}=0, \end{array} \end{array}$$ (3)

where $D_{M,t}^{\varsigma ,\delta }$ is M-TD and *ς* and *δ* are fractional parameters.

In L-FD, the proposed equation takes the following form:

$$\begin{array}{c} \begin{array}{c} N_{hyp,t}^{\varsigma }a_{z}-a_{yyz}+2{\left(aa_{y}\right)}_{z}+2b_{yy}=0,\\ N_{hyp,t}^{\varsigma }b+b_{yy}+2{\left(ab\right)}_{y}=0, \end{array} \end{array}$$ (4)

where $N_{hyp,t}^{\varsigma }$ is L-FD and *ς* is fractional parameter.

In CD, the proposed equation has the following form:

$$\begin{array}{c} \begin{array}{c} D_{c,t}^{\varsigma }a_{z}-a_{yyz}+2{\left(aa_{y}\right)}_{z}+2b_{yy}=0,\\ D_{c,t}^{\varsigma }b+b_{yy}+2{\left(ab\right)}_{y}=0, \end{array} \end{array}$$ (5)

where $D_{c,t}^{\varsigma }$ is CD and *ς* is fractional parameter.

However, a wider range of physical issues required more intricate mathematical differentiation operators. Fractional differentiation [21–27] and the notion of the fractal derivative have been combined to form an innovative differentiation concept. As a result, several mathematicians offered various types of fractional derivatives [28, 29]. The recently proposed derivatives meet a variety of conditions that were previously believed to constitute limitations for fractional derivatives and are utilised to portray various medical circumstances. The conformable fractional derivative definition contains linearity, chain rule, Rolle’s theorem [30], product rule, quotient rule, power rule, and mean value theorem in addition to being fundamental and satisfying the most of the requirements for the ordinary integral derivative. The extended tanh–coth approach and the Jacobi elliptic function method have been used to solve the (4+1)-dimensional fractional Fokas equation (FFE) with a M-truncated derivative, yielding hyperbolic, trigonometric, elliptic, and rational fractional solutions [31]. This study aims to discover soliton wave solutions for the given equation with various derivatives. The four substitute derivatives, which strive to expand the usual derivative by incorporating some natural aspacts, offer a novel approach for various NLPDEs [32].

It has proven possible to solve these non-linear PDEs using a variety of analytical techniques. Instances include the F-expansion technique [33], the Nucci method [34], the RB-sub ODE method [35], the modified auxiliary equation method [36], and many other techniques used to solve PDEs. The Jacobi elliptic functions approach [37] has been used to create a number of multiple solitons for the new coupled Riemann wave equation [18]. The generalised Bernoulli (GB) sub-ODE [38] and the unified methods (UM) [39] are two additional incredibly important techniques that have been used to interpret the given model. To the greatest extent of our knowledge, neither the GB sub-ODE approach nor the UM have ever been used to solve the aforementioned problem. For NLPDEs, reliable travelling wave solutions, peaked soliton solutions, and multi-soliton wave solutions were the initial goals of the GB sub-ODE approach.

UM [40] is a novel method for producing accurate DE solutions. It provides a practical approach for dealing with NLEE results. This effective approach is being used to provide satisfying results and enable the discovery of outcomes for several issues that are cropping up in practical mathematics and physics. Although approximation solution techniques may also be used to construct a wide variety of travelling wave solutions, exact solution techniques are more commonly used in the study of evolution equations.

The structure of the paper is as follows: Section 2 explains the fundamental concepts of derivatives and their features. The transformations and a description of the process are provided in Section 3. The proposed approaches are mathematically described in Section 4. To illustrate how the outcome might be physically understood, Section 5 includes graphics along with the computations. Section 6 provides a few concluding remarks to bring the study to a close.

## 2. Preliminaries

This section discusses the definitions of derivatives as well as their basis characteristics. The beta derivative (BD) is a improved conformable fractional derivative. The BD was initially introduced by Atangana. A single-parameter Mittag–Leffler function [41] that also fulfills the criteria of integer-order calculus is used in an M-fractional derivative (M-FD) that Sousa and Oliveira introduced in 2017. This is why we are going to provide a truncated M-FD type that combines the various fractional derivative types already in existence and also meets the classical properties of integer-order calculus. A unique solution for many FDEs is offered by the conformable derivative (CD), which aims to raise the traditional derivative while satisfying certain natural conditions. These derivatives can be thought of as a natural extension of the classical derivative rather than fractional derivatives. We can see a change in the wave profile by varying the value of the fractional parameter.

### 2.1 Beta derivative and its characteristics

**Definition 1** *The BD is another kind of conformable derivative* [18], *which can be described as*:

$$\begin{array}{c} D_{\beta ,h}^{\varsigma }p\left(h\right)=\underset{\varepsilon \to 0}{\text{lim}}\frac{p\left(h+\varepsilon {\left(h+\frac{1}{\Gamma (\gamma )}\right)}^{1-\varsigma }\right)-p\left(h\right)}{\varepsilon },\;\;0<\varsigma \leq 1. \end{array}$$

The BD has the following characteristics.

-
$D_{\beta ,h}^{\varsigma }\left(m\alpha \left(h\right)+r\xi \left(h\right)\right)=m\;D_{\beta ,h}^{\varsigma }\alpha \left(h\right)+rD_{\beta ,h}^{\varsigma }\xi \left(h\right)$ , ∀*m*, *r* ∈ ℜ.
-
$D_{\beta ,h}^{\varsigma }\left(\alpha \left(h\right)*\xi \left(h\right)\right)=\xi \left(h\right)D_{\beta ,h}^{\varsigma }\alpha \left(h\right)+\alpha \left(h\right)D_{\beta ,h}^{\varsigma }\xi \left(h\right)$ .
-
$D_{\beta ,h}^{\varsigma }\{\frac{\alpha (h)}{\xi (h)}\}=\frac{\xi \left(h\right)D_{\beta ,h}^{\varsigma }\alpha \left(h\right)-\alpha \left(h\right)D_{\beta ,h}^{\varsigma }\xi \left(h\right)}{\xi^{2}\left(h\right)}$ .
- A constant has a zero BD.

$D_{\beta ,h}^{\varsigma }\left(u\right)=0$ , for any *u* constant.

### 2.2 M-truncated derivative and its characteristics

**Definition 2** *For the function p* : [0, ∞) → *R of order* *ς* ∈ (0, 1), *the M-TD* [20] *is defined, as*

$$D_{M,h}^{\varsigma ,\delta }p\left(h\right)=\frac{\underset{\varepsilon \to 0}{\text{lim}}p\left(hE_{j}^{\delta }\left(\varepsilon h^{-\varsigma }\right)\right)-p\left(h\right)}{\varepsilon },$$

*for h* > 0. *Where* $E_{j}^{\delta }(.),\delta >0$ *is defined as a truncated Mittag-Leffler function with a single parameter as follows*:

$$E_{j}^{\delta }(h)=\sum_{r=0}^{j}\frac{h^{r}}{\Gamma (\delta r+1)}.$$

The M-TD has the following characteristics.

-
$D_{M,h}^{\varsigma ,\delta }\left(m\alpha \left(h\right)+r\xi \left(h\right)\right)=mD_{M,h}^{\sigma ,\delta }d\left(h\right)+rD_{M,h}^{\sigma ,\delta }\xi \left(h\right)$ , ∀*m*, *r* ∈ ℜ.
-
$D_{M,h}^{\varsigma ,\delta }\left(\alpha \left(h\right)*\xi \left(h\right)\right)=\xi \left(h\right)D_{M,h}^{\sigma ,\delta }\alpha \left(h\right)+\alpha \left(h\right)D_{M,h}^{\sigma ,\delta }\xi \left(h\right)$ .
-
$D_{M,h}^{\varsigma ,\delta }\{\frac{\alpha (h)}{\xi (h)}\}=\frac{\xi \left(h\right)D_{M,h}^{\sigma ,\delta }\alpha \left(h\right)-\alpha \left(h\right)D_{M,h}^{\sigma ,\delta }\xi \left(h\right)}{\xi^{2}\left(h\right)}$ .
- A differentiable function *ξ*(*h*) has the following M-TD:
  $$D_{M,h}^{\varsigma ,\delta }\xi \left(h\right)=\frac{h^{1-\varsigma }}{\Gamma (\delta +1)}\frac{d\xi }{dh}.$$

### 2.3 A Fractional Local-Derivative

**Definition 3** *The L-FD is defined as below for every* 0 < *t*:

$$\begin{array}{c} N_{hyp}^{\varsigma }p\left(h\right)=\underset{\varepsilon \to 0}{\text{lim}}\frac{p(h+\varepsilon h^{\frac{1-\varsigma }{2}}sech\left(\left(1-\varsigma \right)h^{\frac{1+\varsigma }{2}}\right))-p\left(h\right)}{\varepsilon },\;\;\;\;\;\;\;\;\;\;0<\varsigma <1. \end{array}$$

*If the* $\underset{h\to {f_{0}}^{+}}{\text{lim}}\;N_{hyp}^{\varsigma }\left(p\left(h\right)\right)$ *exists for p* : [*f*_0_, *f*_1_] → ℜ *with f*_0_ < *h*, *It can be stated that*

$$\begin{array}{c} \underset{h\to {f_{0}}^{+}}{\text{lim}}\;N_{hyp}^{\varsigma }\left(p\left(h\right)\right)=N_{hyp}^{\varsigma }\left(p\left(f_{0}\right)\right), \end{array}$$

*and p is ς-differentiable at f*_0_ *with respect to* $N_{hyp}^{\varsigma }\left(p\left(h\right)\right).$

*The L-FD also satisfies*

$$\begin{array}{c} N_{hyp}^{\varsigma }\left(p\left(h\right)\right)=h^{\frac{1-\varsigma }{2}}sech\left(\left(1-\varsigma \right)h^{\frac{1+\varsigma }{2}}\right)\frac{dp(h)}{dh}, \end{array}$$

*in which p* : [*f*_0_, *f*_1_] → ℜ *is differentiable for f*_0_ < *h*.

Moreover, theorems and characteristics relating to L-FD are discussed in [19]

### 2.4 Conformable derivative

**Definition 4** *For the function p* : [0, ∞) → ℜ, *the CD of the order* *ς is written as*:

$$D_{c,h}^{\varsigma }p\left(h\right)=\underset{\varepsilon \to 0}{\text{lim}}\frac{p\left(h+\varepsilon {\left(h\right)}^{1-\varsigma }\right)-p\left(h\right)}{\varepsilon },\;\;\forall h>0.$$

*If p has ς-differentiability in any interval* (0, *q*) *with q* > 0, *then*

$$D_{c}^{\varsigma }\left(p\left(0\right)\right)=\underset{h\to 0^{+}}{\text{lim}}\;D_{c}^{\varsigma }\left(p\left(h\right)\right),$$

*as soon as the limit of the right hand side occur*.

Moreover, in [42], CD-related theorems and characteristics are explanied.

## 3. Explication of the procedure

In this section, we employ the transformations of four distinct derivatives to convert the partial differential equation into an ordinary differential equation. This allows us to apply the techniques to determine the analytical solutions of the BKK system. Let

$$\begin{array}{c} a(y,z,t)=A(\eta ),\;\;\;\;\;b(y,z,t)=B(\eta ), \end{array}$$ (6)

satisfy the following general equation:

$$T(a,a_{y},a_{z},a_{t},a_{yy},a_{zz},a_{yz},\ldots \ldots )=0,$$

which can be transformed into an O.D.E. as:

$$K(A,A^{\prime },A^{\prime \prime }\ldots \ldots )=0.$$

For the BKK equation, taking into account the wave transformations.

For BD, *η* has the subsequent form

$$\begin{array}{c} \eta =dy+pz+\frac{s}{\varsigma }{\left(t+\frac{1}{\Gamma (\varsigma )}\right)}^{\varsigma }. \end{array}$$ (7)

For M-TD, *η* has the subsequent form

$$\begin{array}{c} \eta =dy+pz+s\frac{\Gamma (\delta +1)}{\varsigma }t^{\varsigma }. \end{array}$$ (8)

For L-FD, *η* has the subsequent form

$$\begin{array}{c} \eta =dy+pz+\frac{2}{\left(1-\varsigma^{2}\right)}s\;\text{sinh}((1-\varsigma )t^{\frac{1+\varsigma }{2}}). \end{array}$$ (9)

For CD, *η* has the subsequent form

$$\begin{array}{c} \eta =dy+pz+\frac{s}{\varsigma }{\left(t\right)}^{\varsigma }. \end{array}$$ (10)

Where the values of *d*, *p* and, *s* are any constants with *p*, *d* and, *s* ≠ 0. By empolying the transformations of Eq (7), together with Eq’s (8), (9) and (10) we have

$$\begin{array}{c} \begin{array}{c} psA^{\prime \prime }-d^{2}pA^{\prime \prime \prime }+2dp{\left(AA^{\prime }\right)}^{\prime }+2d^{2}B^{\prime \prime }=0,\\ sB^{\prime }+d^{2}B^{\prime \prime }+2d{\left(AB\right)}^{\prime }=0. \end{array} \end{array}$$ (11)

Integrating twice the first equation of Eq (11) and taking the integration constants to zero, we have

$$\begin{array}{c} B\left(\eta \right)=\frac{-psA}{2d^{2}}+\frac{pA^{\prime }}{2}-\frac{pA^{2}}{2d}. \end{array}$$ (12)

Now that we have integrated the second equation of Eq (11) and taking the integration constant to zero, we have

$$\begin{array}{c} sB+d^{2}B^{\prime }+2d\left(AB\right)=0. \end{array}$$ (13)

Substitute Eq (12) into Eq (13), we have

$$\begin{array}{c} d_{1}A^{\prime \prime }+d_{2}A^{3}+d_{3}A^{2}+d_{4}A=0, \end{array}$$ (14)

where $d_{1}=\frac{pd^{2}}{2}$, *d*_2_ = −*p*, $d_{3}=\frac{-3ps}{2d}$, $d_{4}=\frac{-ps^{2}}{2d^{2}},$ and $A^{\prime }=\frac{dA}{d\eta }.$

## 4. Mathematical description of the proposed methods

In this section, we employ a step-by-step procedure to solve the ODE of the BKK system and find out the constant values to provide a physical description for analytical solutions.

### 4.1 Unified method

The suggested expansion can be found in the general solution as follows [39]:

$$\begin{array}{c} A\left(\eta \right)=\psi_{0}+\sum_{q=1}^{n}[\psi_{q}{\left(\mu \right)}^{q}+\phi_{q}{\left(\mu \right)}^{-q}], \end{array}$$ (15)

where *ψ*_*q*_ and *ϕ*_*q*_ are arbitrary constants found later. Also *μ*(*η*) can be obtained using the Ricatti differential equation.

$$\begin{array}{c} \mu^{\prime }\left(\eta \right)=\mu^{2}\left(\eta \right)+\vartheta , \end{array}$$ (16)

where $\mu^{\prime }=\frac{d\mu }{d\eta }$. Solutions for the Eq (16) are discussed below.

**Family 1** When *ϑ* < 0.

$\mu \left(\eta \right)=\frac{\sqrt{-(H^{2}+R^{2})\vartheta }-H\sqrt{-\vartheta }\text{cosh}(2\sqrt{-\vartheta }(\eta +B))}{H\text{sinh}(2\sqrt{-\vartheta }(\eta +B))+R},$ *or* $\mu \left(\eta \right)=\frac{-\sqrt{-(H^{2}+R^{2})\vartheta }-H\sqrt{-\vartheta }\text{cosh}(2\sqrt{-\vartheta }(\eta +B))}{H\text{sinh}(2\sqrt{-\vartheta }(\eta +B))+R},$

$\mu \left(\eta \right)=\sqrt{-\vartheta }+\frac{-2H\sqrt{-\vartheta }}{H+\text{cosh}(2\sqrt{-\vartheta }(\eta +B))-\text{sinh}(2\sqrt{-\vartheta }(\eta +B))},$ *or* $\mu \left(\eta \right)=-\sqrt{-\vartheta }+\frac{-2H\sqrt{-\vartheta }}{H+\text{cosh}(2\sqrt{-\vartheta }(\eta +B))-\text{sinh}(2\sqrt{-\vartheta }(\eta +B))}.$

**Family 2** When *ϑ* > 0.

$\mu \left(\eta \right)=\frac{\sqrt{(H^{2}+R^{2})\vartheta }-H\sqrt{\vartheta }\text{cos}(2\sqrt{\vartheta }(\eta +B))}{H\text{sin}(2\sqrt{\vartheta }(\eta +B))+R}$ , *or* $\mu \left(\eta \right)=\frac{-\sqrt{(H^{2}+R^{2})\vartheta }-H\sqrt{\vartheta }\text{cos}(2\sqrt{\vartheta }(\eta +B))}{H\text{sin}(2\sqrt{\vartheta }(\eta +B))+R}$,

$\mu \left(\eta \right)=i\sqrt{\vartheta }+\frac{-2Hi\sqrt{\vartheta }}{H+\text{cos}(2\sqrt{\vartheta }(\eta +B))-\iota \text{sin}(2\sqrt{\vartheta }(\eta +B))},$ *or* $\mu \left(\eta \right)=-i\sqrt{\vartheta }+\frac{-2Hi\sqrt{\vartheta }}{H+\text{cos}(2\sqrt{\vartheta }(\eta +B))-\iota \text{sin}(2\sqrt{\vartheta }(\eta +B))}$ .

**Family 3** When *ϑ* = 0.

$\mu \left(\eta \right)=-\frac{1}{\eta +B},$

where *H* and *R* are real arbitrary constants, and *B* is any arbitrary constant.

The homogeneous balancing principle is used to balance the highest order derivative *A*″ and highest order nonlinear term *A*^3^ in Eq (14), which leads to *n* = 1.

$$\begin{array}{c} A(\eta )=\psi_{0}+pomcol\psi_{1}\left(\mu \right)+\phi_{1}{\left(\mu \right)}^{-1}. \end{array}$$

When each coefficient of *μ*(*η*) is set to zero, the following set of algebraic equations results:

$$\begin{array}{c} \mu {\left(\eta \right)}^{3}=2d_{1}\psi_{1}+d_{2}\psi_{1}^{3},\\ \mu {\left(\eta \right)}^{2}=\left(d_{3}+3d_{2}\psi_{0}\right)\psi_{1}^{2},\\ \mu {\left(\eta \right)}^{1}=\psi_{1}\left(2\vartheta d_{1}+d_{4}+2d_{3}\psi_{0}+3d_{2}\left(\psi_{0}^{2}+\phi_{1}\psi_{1}\right)\right),\\ \mu {\left(\eta \right)}^{0}=d_{4}\psi_{0}+d_{3}\psi_{0}^{2}+d_{2}\psi_{0}^{3}+2d_{3}\phi_{1}\psi_{1}+6d_{2}\phi_{1}\psi_{0}\psi_{1},\\ \mu {\left(\eta \right)}^{-1}=\phi_{1}\left(2\vartheta d_{1}+d_{4}+2d_{3}\psi_{0}+3d_{2}\left(\psi_{0}^{2}+\phi_{1}\psi_{1}\right)\right),\\ \mu {\left(\eta \right)}^{-2}=\phi_{1}^{2}\left(d_{3}+3d_{2}\psi_{0}\right),\\ \mu {\left(\eta \right)}^{-3}=2\vartheta^{2}d_{1}\phi_{1}+d_{2}\phi_{1}^{3}. \end{array}$$

The equations above being resolved result in the following families:

**Family 1** When

$\psi_{0}=-\frac{d_{3}}{3d_{2}},\psi_{1}=0,\phi_{1}=-\frac{i\sqrt{2}\vartheta \sqrt{d_{1}}}{\sqrt{d_{2}}}.$

The following cases will occur:

-
$a_{1,1}(y,z,t)=-\frac{s}{2d}-\frac{i\sqrt{d^{2}p}\vartheta (R+H\text{sinh}(2\sqrt{-\vartheta }(B+\eta )))}{\sqrt{-p}(\sqrt{(-H^{2}-R^{2})\vartheta }-H\sqrt{-\vartheta }\text{cosh}(2\sqrt{-\vartheta }(B+\eta )))},$

-
$a_{1,2}(y,z,t)=-\frac{s}{2d}-\frac{i\sqrt{d^{2}p}\vartheta (R+H\text{sinh}(2\sqrt{-\vartheta }(B+\eta )))}{\sqrt{-p}(-\sqrt{(-H^{2}-R^{2})\vartheta }-H\sqrt{-\vartheta }\text{cosh}(2\sqrt{-\vartheta }(B+\eta )))},$

-
$a_{1,3}(y,z,t)=-\frac{s}{2d}-\frac{i\sqrt{d^{2}p}\vartheta }{\sqrt{-p}(\sqrt{-\vartheta }-\frac{2H\sqrt{-\vartheta }}{H+\text{cosh}(2\sqrt{-\vartheta }(B+\eta ))-\text{sinh}(2\sqrt{-\vartheta }(B+\eta ))})},$

-
$a_{1,4}(y,z,t)=-\frac{s}{2d}-\frac{i\sqrt{d^{2}p}\vartheta }{\sqrt{-p}(-\sqrt{-\vartheta }-\frac{2H\sqrt{-\vartheta }}{H+\text{cosh}(2\sqrt{-\vartheta }(B+\eta ))-\text{sinh}(2\sqrt{-\vartheta }(B+\eta ))})},$

-
$a_{1,5}(y,z,t)=-\frac{s}{2d}-\frac{i\sqrt{d^{2}p}\vartheta (R+H\;\text{sin}(2\sqrt{\vartheta }(B+\eta )))}{\sqrt{-p}(\sqrt{(H^{2}+R^{2})\vartheta }-H\sqrt{\vartheta }\;\text{cos}(2\sqrt{\vartheta }(B+\eta )))},$

-
$a_{1,6}(y,z,t)=-\frac{s}{2d}-\frac{i\sqrt{d^{2}p}\vartheta (R+H\;\text{sin}(2\sqrt{\vartheta }(B+\eta )))}{\sqrt{-p}(-\sqrt{(H^{2}-R^{2})\vartheta }-H\sqrt{\vartheta }\;\text{cos}(2\sqrt{\vartheta }(B+\eta )))},$

-
$a_{1,7}(y,z,t)=-\frac{s}{2d}-\frac{i\sqrt{d^{2}p}\vartheta }{\sqrt{-p}(i\sqrt{\vartheta }-\frac{2iH\sqrt{-\vartheta }}{H+\;\text{cos}[2\sqrt{\vartheta }(B+\eta )]-i\;\text{sin}[2\sqrt{\vartheta }(B+\eta )]})},$

-
$a_{1,8}(y,z,t)=-\frac{s}{2d}-\frac{i\sqrt{d^{2}p}\vartheta }{\sqrt{-p}(-i\sqrt{\vartheta }+\frac{2iH\sqrt{\vartheta }}{H+\;\text{cos}[2\sqrt{\vartheta }(B+\eta )]-i\;\text{sin}[2\sqrt{\vartheta }(B+\eta )]})}.$

**Family 2** When

$\psi_{0}=-\frac{d_{3}}{3d_{2}},\psi_{1}=-\frac{i\sqrt{2}\sqrt{d_{1}}}{\sqrt{d_{2}}},\phi_{1}=-\frac{i\sqrt{2}\vartheta \sqrt{d_{1}}}{\sqrt{d_{2}}}.$

The following cases will occur:

-
$a_{2,1}(y,z,t)=\begin{array}{c} -\frac{s}{2d}-\frac{i\sqrt{d^{2}p}(\sqrt{(-H^{2}-R^{2})\vartheta }-H\sqrt{-\vartheta }\;\text{cosh}(2\sqrt{-\vartheta }(B+\eta )))}{\sqrt{-p}(R+H\;\text{sinh}(2\sqrt{-\vartheta }(B+\eta )))}-\\ \frac{i\sqrt{d^{2}p}\vartheta (R+H\;\text{sinh}(2\sqrt{-\vartheta }(B+\eta )))}{\sqrt{-p}(\sqrt{(-H^{2}-R^{2})\vartheta }-H\sqrt{-\vartheta }\;\text{cosh}(2\sqrt{-\vartheta }(B+\eta )))}, \end{array}$

-
$a_{2,2}(y,z,t)=\begin{array}{c} -\frac{s}{2d}-\frac{i\sqrt{d^{2}p}(-\sqrt{(-H^{2}-R^{2})\vartheta }-H\sqrt{-\vartheta }\;\text{cosh}(2\sqrt{-\vartheta }(B+\eta )))}{\sqrt{-p}(R+H\;\text{sinh}(2\sqrt{-\vartheta }(B+\eta )))}-\\ -\frac{i\sqrt{d^{2}p}v(R+H\;\text{sinh}(2\sqrt{-\vartheta }(B+\eta )))}{\sqrt{-p}(-\sqrt{(-H^{2}-R^{2})\vartheta }-H\sqrt{-v}\;\text{cosh}(2\sqrt{-\vartheta }(B+\eta )))}, \end{array}$

-
$a_{2,3}(y,z,t)=\begin{array}{c} -\frac{s}{2d}-\frac{i\sqrt{d^{2}p}\vartheta }{\sqrt{-p}(\sqrt{-\vartheta }-\frac{2H\sqrt{-\vartheta }}{H+\;\text{cosh}(2\sqrt{-\vartheta }(B+\eta ))-\;\text{sinh}(2\sqrt{-\vartheta }(B+\eta ))})}-\\ \frac{i\sqrt{d^{2}p}(\sqrt{-\vartheta }-\frac{2H\sqrt{-\vartheta }}{H+\;\text{cosh}(2\sqrt{-\vartheta }(B+\eta ))-\;\text{sinh}(2\sqrt{-\vartheta }(B+\eta ))})}{\sqrt{-p}}, \end{array}$

-
$a_{2,4}(y,z,t)=\begin{array}{c} -\frac{s}{2d}-\frac{i\sqrt{d^{2}p}\vartheta }{\sqrt{-p}(-\sqrt{-\vartheta }-\frac{2H\sqrt{-\vartheta }}{H+\;\text{cosh}(2\sqrt{-v}(B+\eta ))-\;\text{sinh}(2\sqrt{-\vartheta }(B+\eta ))})}-\\ \frac{i\sqrt{d^{2}p}(-\sqrt{-\vartheta }-\frac{2H\sqrt{-v}}{H+\;\text{cosh}(2\sqrt{-\vartheta }(B+\eta ))-\;\text{sinh}(2\sqrt{-\vartheta }(B+\eta ))})}{\sqrt{-p}}, \end{array}$

-
$a_{2,5}(y,z,t)=\begin{array}{c} -\frac{s}{2d}-\frac{i\sqrt{d^{2}p}(\sqrt{(H^{2}+R^{2})\vartheta }-H\sqrt{\vartheta }\;\text{cos}(2\sqrt{\vartheta }(B+\eta )))}{\sqrt{-p}(R+H\;\text{sin}(2\sqrt{\vartheta }(B+\eta )))}-\\ \frac{i\sqrt{d^{2}p}\vartheta (R+H\;\text{sin}(2\sqrt{\vartheta }(B+\eta )))}{\sqrt{-p}(\sqrt{(H^{2}+R^{2})\vartheta }-H\sqrt{\vartheta }\;\text{cos}(2\sqrt{\vartheta }(B+\eta )))}, \end{array}$

-
$a_{2,6}(y,z,t)=\begin{array}{c} -\frac{s}{2d}-\frac{i\sqrt{d^{2}p}(-\sqrt{(H^{2}-R^{2})\vartheta }-H\sqrt{\vartheta }\;\text{cos}(2\sqrt{\vartheta }(B+\eta )))}{\sqrt{-p}(R+H\;\text{sin}(2\sqrt{\vartheta }(B+\eta )))}-\\ \frac{i\sqrt{d^{2}p}\vartheta (R+H\;\text{sin}(2\sqrt{\vartheta }(B+\eta )))}{\sqrt{-p}(-\sqrt{(H^{2}-R^{2})\vartheta }-H\sqrt{\vartheta }\;\text{cos}(2\sqrt{\vartheta }(B+\eta )))}, \end{array}$

-
$a_{2,7}(y,z,t)=\begin{array}{c} -\frac{s}{2d}-\frac{i\sqrt{d^{2}p}\vartheta }{\sqrt{-p}(i\sqrt{\vartheta }-\frac{2iH\sqrt{-\vartheta }}{H+\;\text{cos}(2\sqrt{\vartheta }(B+\eta ))-i\;\text{sin}(2\sqrt{\vartheta }(B+\eta ))})}-\\ \frac{i\sqrt{d^{2}p}(i\sqrt{\vartheta }-\frac{2iH\sqrt{-\vartheta }}{H+\;\text{cos}(2\sqrt{\vartheta }(B+\eta ))-i\;\text{sin}(2\sqrt{\vartheta }(B+\eta ))})}{\sqrt{-p}}, \end{array}$

-
$a_{2,8}(y,z,t)=\begin{array}{c} -\frac{s}{2d}-\frac{i\sqrt{d^{2}p}\vartheta }{\sqrt{-p}(-i\sqrt{\vartheta }-\frac{2iH\sqrt{-\vartheta }}{H+\;\text{cos}(2\sqrt{\vartheta }(B+\eta ))-i\;\text{sin}(2\sqrt{\vartheta }(B+\eta ))})}-\\ \frac{i\sqrt{d^{2}p}(-i\sqrt{\vartheta }-\frac{2iH\sqrt{-\vartheta }}{H+\;\text{cos}(2\sqrt{\vartheta }(B+\eta ))-i\;\text{sin}(2\sqrt{\vartheta }(B+\eta ))})}{\sqrt{-p}}. \end{array}$

**Family 3** When

$\psi_{0}=-\frac{d_{3}}{3d_{2}},\psi_{1}=-\frac{i\sqrt{2}\sqrt{d_{1}}}{\sqrt{d_{2}}},\phi_{1}=0.$

The following cases will occur:

-
$a_{3,1}(y,z,t)=-\frac{s}{2d}-\frac{i\sqrt{d^{2}p}(\sqrt{(-H^{2}-R^{2})\vartheta }-H\sqrt{-\vartheta }\;\text{cosh}(2\sqrt{-\vartheta }(B+\eta )))}{\sqrt{-p}(R+H\;\text{sinh}(2\sqrt{-\vartheta }(B+\eta )))},$

-
$a_{3,2}(y,z,t)=-\frac{s}{2d}-\frac{i\sqrt{d^{2}p}(-\sqrt{(-H^{2}-R^{2})\vartheta }-H\sqrt{-\vartheta }\;\text{cosh}(2\sqrt{-\vartheta }(B+\eta )))}{\sqrt{-p}(R+H\;\text{sinh}(2\sqrt{-\vartheta }(B+\eta )))},$

-
$a_{3,3}(y,z,t)=-\frac{s}{2d}-\frac{i\sqrt{d^{2}p}(\sqrt{-\vartheta }-\frac{2H\sqrt{-\vartheta }}{H+\;\text{cosh}(2\sqrt{-\vartheta }(B+\eta ))-\;\text{sinh}(2\sqrt{-\vartheta }(B+\eta ))})}{\sqrt{-p}},$

-
$a_{3,4}(y,z,t)=-\frac{s}{2d}-\frac{i\sqrt{d^{2}p}(-\sqrt{-\vartheta }-\frac{2H\sqrt{-\vartheta }}{H+\;\text{cosh}(2\sqrt{-\vartheta }(B+\eta ))-\;\text{sinh}(2\sqrt{-\vartheta }(B+\eta ))})}{\sqrt{-p}},$

-
$a_{3,5}(y,z,t)=-\frac{s}{2d}-\frac{i\sqrt{d^{2}p}(\sqrt{(H^{2}+R^{2})\vartheta }-H\sqrt{\vartheta }\;\text{cos}(2\sqrt{\vartheta }(B+\eta )))}{\sqrt{-p}(R+H\;\text{sin}(2\sqrt{\vartheta }(B+\eta )))},$

-
$a_{3,6}(y,z,t)=-\frac{s}{2d}-\frac{i\sqrt{d^{2}p}(-\sqrt{(H^{2}+R^{2})\vartheta }-H\sqrt{\vartheta }\;\text{cos}(2\sqrt{\vartheta }(B+\eta )))}{\sqrt{-p}(R+H\;\text{sin}(2\sqrt{\vartheta }(B+\eta )))},$

-
$a_{3,7}(y,z,t)=-\frac{s}{2d}-\frac{i\sqrt{d^{2}p}(i\sqrt{\vartheta }-\frac{2iH\sqrt{-\vartheta }}{H+\;\text{cos}(2\sqrt{\vartheta }(B+\eta ))-i\;\text{sin}(2\sqrt{\vartheta }(B+\eta ))})}{\sqrt{-p}},$

-
$a_{3,8}(y,z,t)=-\frac{s}{2d}-\frac{i\sqrt{d^{2}p}(-i\sqrt{\vartheta }-\frac{2iH\sqrt{-\vartheta }}{H+\;\text{cos}(2\sqrt{\vartheta }(B+\eta ))-i\;\text{sin}(2\sqrt{\vartheta }(B+\eta ))})}{\sqrt{-p}}.$

**Family 4** When

$\psi_{0}=-\frac{d_{3}}{3d_{2}},\psi_{1}=\frac{i\sqrt{2}\sqrt{d_{1}}}{\sqrt{d_{2}}},\phi_{1}=-\frac{i\sqrt{2}\vartheta \sqrt{d_{1}}}{\sqrt{d_{2}}}.$

The following cases will occur:

-
$a_{4,1}(y,z,t)=\begin{array}{c} -\frac{s}{2d}+\frac{i\sqrt{d^{2}p}(\sqrt{(-H^{2}-R^{2})\vartheta }-H\sqrt{-\vartheta }\;\text{cosh}(2\sqrt{-\vartheta }(B+\eta )))}{\sqrt{-p}(R+H\;\text{sinh}[2\sqrt{-\vartheta }(B+\eta [y,z,t])])}-\\ \frac{i\sqrt{d^{2}p}\vartheta (R+H\;\text{sinh}(2\sqrt{-\vartheta }(B+\eta )))}{\sqrt{-p}(\sqrt{(-H^{2}-R^{2})\vartheta }-H\sqrt{-\vartheta }\;\text{cosh}(2\sqrt{-\vartheta }(B+\eta )))}, \end{array}$

-
$a_{4,2}(y,z,t)=\begin{array}{c} -\frac{s}{2d}+\frac{i\sqrt{d^{2}p}(-\sqrt{(-H^{2}-R^{2})\vartheta }-H\sqrt{-\vartheta }\;\text{cosh}(2\sqrt{-\vartheta }(B+\eta )))}{\sqrt{-p}(R+H\;\text{sinh}[2\sqrt{-\vartheta }(B+\eta [y,z,t])])}-\\ \frac{i\sqrt{d^{2}p}\vartheta (R+H\;\text{sinh}(2\sqrt{-\vartheta }(B+\eta )))}{\sqrt{-p}(-\sqrt{(-H^{2}-R^{2})\vartheta }-H\sqrt{-\vartheta }\;\text{cosh}(2\sqrt{-\vartheta }(B+\eta )))}, \end{array}$

-
$a_{4,3}(y,z,t)=\begin{array}{c} -\frac{s}{2d}-\frac{i\sqrt{d^{2}p}\vartheta }{\sqrt{-p}(\sqrt{-\vartheta }-\frac{2H\sqrt{-\vartheta }}{H+\;\text{cosh}(2\sqrt{-\vartheta }(B+\eta ))-\;\text{sinh}(2\sqrt{-\vartheta }(B+\eta ))})}+\\ \frac{i\sqrt{d^{2}p}(\sqrt{-\vartheta }-\frac{2H\sqrt{-\vartheta }}{H+\;\text{cosh}(2\sqrt{-\vartheta }(B+\eta ))-\;\text{sinh}(2\sqrt{-\vartheta }(B+\eta ))})}{\sqrt{-p}}, \end{array}$

-
$a_{4,4}(y,z,t)=\begin{array}{c} -\frac{s}{2d}-\frac{i\sqrt{d^{2}p}\vartheta }{\sqrt{-p}(-\sqrt{-\vartheta }-\frac{2H\sqrt{-\vartheta }}{H+\;\text{cosh}(2\sqrt{-\vartheta }(B+\eta ))-\;\text{sinh}(2\sqrt{-\vartheta }(B+\eta ))})}+\\ \frac{i\sqrt{d^{2}p}(-\sqrt{-\vartheta }-\frac{2H\sqrt{-\vartheta }}{H+\;\text{cosh}(2\sqrt{-\vartheta }(B+\eta ))-\;\text{sinh}(2\sqrt{-\vartheta }(B+\eta ))})}{\sqrt{-p}}, \end{array}$

-
$a_{4,5}(y,z,t)=\begin{array}{c} -\frac{s}{2d}+\frac{i\sqrt{d^{2}p}(\sqrt{(H^{2}+R^{2})\vartheta }-H\sqrt{\vartheta }\;\text{cos}(2\sqrt{\vartheta }(B+\eta )))}{\sqrt{-p}(R+H\;\text{sin}(2\sqrt{\vartheta }(B+\eta )))}-\\ \frac{i\sqrt{d^{2}p}\vartheta (R+H\;\text{sin}(2\sqrt{\vartheta }(B+\eta )))}{\sqrt{-p}(\sqrt{(H^{2}+R^{2})\vartheta }-H\sqrt{\vartheta }\;\text{cos}(2\sqrt{\vartheta }(B+\eta )))}, \end{array}$

-
$a_{4,6}(y,z,t)=\begin{array}{c} -\frac{s}{2d}+\frac{i\sqrt{d^{2}p}(-\sqrt{(H^{2}+R^{2})\vartheta }-H\sqrt{\vartheta }\;\text{cos}(2\sqrt{\vartheta }(B+\eta )))}{\sqrt{-p}(R+H\;\text{sin}(2\sqrt{\vartheta }(B+\eta )))}-\\ \frac{i\sqrt{d^{2}p}\vartheta (R+H\;\text{sin}(2\sqrt{\vartheta }(B+\eta )))}{\sqrt{-p}(-\sqrt{(H^{2}+R^{2})\vartheta }-H\sqrt{\vartheta }\;\text{cos}(2\sqrt{\vartheta }(B+\eta )))}, \end{array}$

-
$a_{4,7}(y,z,t)=\begin{array}{c} -\frac{s}{2d}-\frac{i\sqrt{d^{2}p}\vartheta }{\sqrt{-p}(i\sqrt{\vartheta }-\frac{2iH\sqrt{-\vartheta }}{H+\;\text{cos}(2\sqrt{\vartheta }(B+\eta ))-i\;\text{sin}(2\sqrt{\vartheta }(B+\eta ))})}+\\ \frac{i\sqrt{d^{2}p}(i\sqrt{\vartheta }-\frac{2iH\sqrt{-\vartheta }}{H+\;\text{cos}(2\sqrt{\vartheta }(B+\eta ))-i\;\text{sin}(2\sqrt{\vartheta }(B+\eta ))})}{\sqrt{-p}}, \end{array}$

-
$a_{4,8}(y,z,t)=\begin{array}{c} -\frac{s}{2d}-\frac{i\sqrt{d^{2}p}\vartheta }{\sqrt{-p}(-i\sqrt{\vartheta }-\frac{2iH\sqrt{-\vartheta }}{H+\;\text{cos}(2\sqrt{\vartheta }(B+\eta ))-i\;\text{sin}(2\sqrt{\vartheta }(B+\eta ))})}+\\ \frac{i\sqrt{d^{2}p}(-i\sqrt{\vartheta }-\frac{2iH\sqrt{-\vartheta }}{H+\;\text{cos}(2\sqrt{\vartheta }(B+\eta ))-i\;\text{sin}(2\sqrt{\vartheta }(B+\eta ))})}{\sqrt{-p}}. \end{array}$

Now, by using the values of *A*(*η*) from the above equations, we can find the values of *b*(*y*, *z*, *t*) for each of the cases mentioned above.

$$\begin{array}{c} b\left(y,z,t\right)=\frac{-psA}{2d^{2}}+\frac{pA^{\prime }}{2}-\frac{pA^{2}}{2d}. \end{array}$$

### 4.2 GB sub-ODE method

The general solution has the given expansion in the form of the GB sub-ODE method [43] as follows:

$$\begin{array}{c} A\left(\eta \right)=\sum_{q=0}^{n}[\psi_{q}{\left(\mu \right)}^{q}], \end{array}$$ (17)

where *ψ*_*q*_ are arbitrary constants. and *μ* = *μ*(*η*) satisfies the following equation:

$$\begin{array}{c} \mu^{\prime }=\alpha \mu^{2}-\sigma \mu , \end{array}$$ (18)

where *α* ≠ 0, Eq (18) is a specific type of Bernoulli equation, and we can find the solution as

$$\begin{array}{c} \mu \left(\eta \right)=-\frac{\sigma }{2\alpha }(\text{tanh}(\frac{\sigma }{2}\eta )-1),\;\;\text{or}\;\;\mu \left(\eta \right)=-\frac{\sigma }{2\alpha }(\text{coth}(\frac{\sigma }{2}\eta )-1). \end{array}$$

Balancing the order of *A*″ and *A*^3^ appearing in Eq (14), we have n = 1. Consequently, the solution to Eq (14) assumes the following form in accordance with the sub-ODE technique rule.

$$\begin{array}{c} A(\eta )=\psi_{0}+\psi_{1}\mu . \end{array}$$ (19)

The following set of algebraic equations produced by putting Eqs (18) and (19) into Eq (14), combining all the terms to the similar power of *μ*(*η*) and setting each exponent to zero is as follows:

$$\begin{array}{c} \mu {\left(\eta \right)}^{0}=d_{4}\psi_{0}+d_{3}\psi_{0}^{2}+d_{2}\psi_{0}^{3},\\ \;\mu {\left(\eta \right)}^{1}=(\sigma^{2}d_{1}+d_{4}+\psi_{0}(2d_{3}+3d_{2}\psi_{0}))\psi_{1},\\ \;\mu {\left(\eta \right)}^{2}=\psi_{1}(-3\alpha \sigma d_{1}+(d_{3}+3d_{2}\psi_{0})\psi_{1}),\\ \mu {\left(\eta \right)}^{3}=2\alpha^{2}d_{1}\psi_{1}+d_{2}\psi_{1}^{3}. \end{array}$$

**Set 1** When

$\psi_{0}=\frac{i(3\sqrt{2}\sigma \sqrt{d_{1}}\sqrt{d_{2}}+2id_{3})}{6d_{2}},\psi_{1}=-\frac{i\sqrt{2}\alpha \sqrt{d_{1}}}{\sqrt{d_{2}}}.$

-
$a_{1,1}(y,z,t)=-\frac{i(-\frac{3ips}{d}+3\sqrt{-p}\sqrt{d^{2}p}\sigma )}{6p}+\frac{i\sqrt{d^{2}p}\sigma (-1+\text{tanh}(\frac{1}{2}\sigma \eta ))}{2\sqrt{-p}},$

-
$b_{1,1}(y,z,t)=\begin{array}{c} \frac{ip\sqrt{d^{2}p}\sigma^{2}Sech{\left(\frac{\sigma \eta }{2}\right)}^{2}}{8\sqrt{-p}}-\frac{ps(-\frac{i(-\frac{3ips}{d}+3\sqrt{-p}\sqrt{d^{2}p}\sigma )}{6p}+\frac{i\sqrt{d^{2}p}\sigma (-1+\text{tanh}(\frac{\sigma \eta }{2}))}{2\sqrt{-p}})}{2d^{2}}-\\ \frac{p{\left(-\frac{i(-\frac{3ips}{d}+3\sqrt{-p}\sqrt{d^{2}p}\sigma )}{6p}+\frac{i\sqrt{d^{2}p}\sigma (-1+\text{tanh}(\frac{\sigma \eta }{2}))}{2\sqrt{-p}}\right)}^{2}}{2d}, \end{array}$

-
$a_{1,2}(y,z,t)=-\frac{i(-\frac{3ips}{d}+3\sqrt{-p}\sqrt{d^{2}p}\sigma )}{6p}+\frac{i\sqrt{d^{2}p}\sigma (-1+\text{coth}(\frac{1}{2}\sigma \eta ))}{2\sqrt{-p}},$

-
$b_{1,2}(y,z,t)=\begin{array}{c} -\frac{ps(-\frac{i(-\frac{3ips}{d}+3\sqrt{-p}\sqrt{d^{2}p}\sigma )}{6p}+\frac{i\sqrt{d^{2}p}\sigma (-1+\text{coth}(\frac{\sigma \eta }{2}))}{2\sqrt{-p}})}{2d^{2}}-\\ \frac{p{\left(-\frac{i(-\frac{3ips}{d}+3\sqrt{-p}\sqrt{d^{2}p}\sigma )}{6p}+\frac{i\sqrt{d^{2}p}\sigma (-1+\text{coth}(\frac{\sigma \eta }{2}))}{2\sqrt{-p}}\right)}^{2}}{2d}-\frac{ip\sqrt{d^{2}p}\sigma^{2}Csch{\left(\frac{\sigma \eta }{2}\right)}^{2}}{8\sqrt{-p}}. \end{array}$

**Set 2** When

$\psi_{0}=-\frac{i(3\sqrt{2}\sigma \sqrt{d_{1}}\sqrt{d_{2}}+2id_{3})}{6d_{2}},\psi_{1}=\frac{i\sqrt{2}\alpha \sqrt{d_{1}}}{\sqrt{d_{2}}}.$

-
$a_{2,1}(y,z,t)=\frac{i(-\frac{3ips}{d}+3\sqrt{-p}\sqrt{d^{2}p}\sigma )}{6p}-\frac{i\sqrt{d^{2}p}\sigma (-1+\text{tanh}(\frac{1}{2}\sigma \eta ))}{2\sqrt{-p}},$

-
$b_{2,1}(y,z,t)=\begin{array}{c} -\frac{ip\sqrt{d^{2}p}\sigma^{2}Sech{\left(\frac{\sigma \eta }{2}\right)}^{2}}{8\sqrt{-p}}-\frac{ps(\frac{i(-\frac{3ips}{d}+3\sqrt{-p}\sqrt{d^{2}p}\sigma )}{6p}-\frac{i\sqrt{d^{2}p}\sigma (-1+\text{tanh}(\frac{\sigma \eta }{2}))}{2\sqrt{-p}})}{2d^{2}}-\\ \frac{p{\left(\frac{i(-\frac{3ips}{d}+3\sqrt{-p}\sqrt{d^{2}p}\sigma )}{6p}-\frac{i\sqrt{d^{2}p}\sigma (-1+\text{tanh}(\frac{\sigma \eta }{2}))}{2\sqrt{-p}}\right)}^{2}}{2d}, \end{array}$

-
$a_{2,2}(y,z,t)=\frac{i(-\frac{3ips}{d}+3\sqrt{-p}\sqrt{d^{2}p}\sigma )}{6p}-\frac{i\sqrt{d^{2}p}\sigma (-1+\text{coth}(\frac{1}{2}\sigma \eta ))}{2\sqrt{-p}},$

-
$b_{2,2}(y,z,t)=\begin{array}{c} -\frac{ps(\frac{i(-\frac{3ips}{d}+3\sqrt{-p}\sqrt{d^{2}p}\sigma )}{6p}-\frac{i\sqrt{d^{2}p}\sigma (-1+\text{coth}(\frac{\sigma \eta }{2}))}{2\sqrt{-p}})}{2d^{2}}-\\ \frac{p{\left(\frac{i(-\frac{3ips}{d}+3\sqrt{-p}\sqrt{d^{2}p}\sigma )}{6p}-\frac{i\sqrt{d^{2}p}\sigma (-1+\text{coth}(\frac{\sigma \eta }{2}))}{2\sqrt{-p}}\right)}^{2}}{2d}+\frac{ip\sqrt{d^{2}p}\sigma^{2}Csch{\left(\frac{\sigma \eta }{2}\right)}^{2}}{8\sqrt{-p}}. \end{array}$

## 5. Graphical portrayal and interpretation

**Fig 1:**
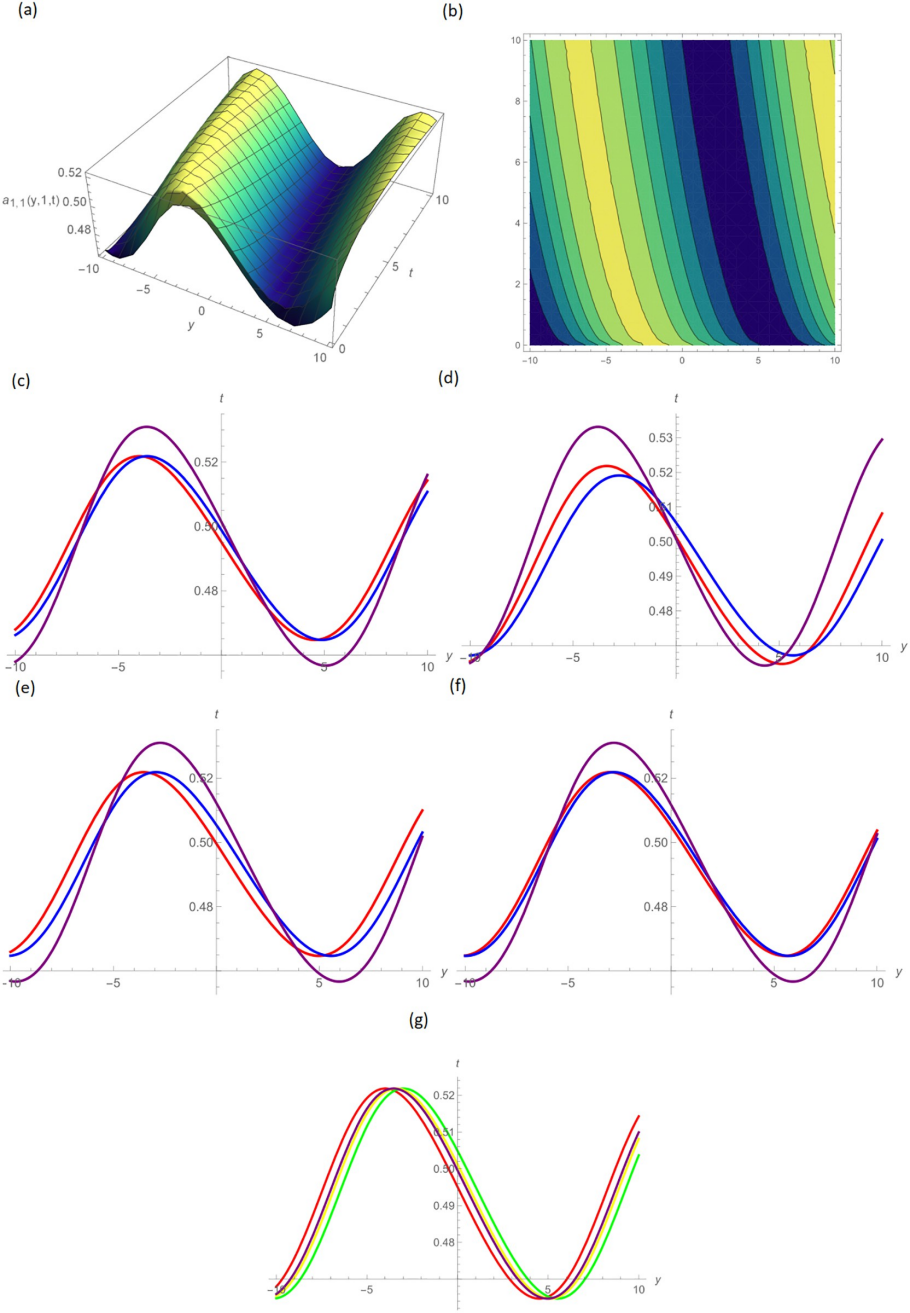
Single wave type shape solution of *a*_1,1_(*y*, 1, *t*) by UM, (a) 3D plot, (b): Contour plot, when H = 0.5, R = 3.5, *ϑ* = 4, B = 0.5, d = 0.1, s = 0.09, p = 1.5, (c): BD in 2D at different values of *ς*=0.45(red),*ς*=0.65(blue),*ς*=0.9 (purple), (d): M-TD in 2D at different values of *ς*=0.5,*δ*=0.25(red),*ς*=0.75, *δ*=0.5(blue),*ς*=0.9,*δ*=0.75(purple), (e): CD in 2D at different values of *ς*=0.45,(red),*ς*=0.65(blue),*ς*=0.9 (purple), (f): L-FD in 2D at different values of *ς*=0.5(red),*ς*=0.75(blue),*ς*=0.9(purple), (g): A comparison of BD(red), L-FD(green), M-TD(yellow) and CD(purple) at *ς* = 0.5.

**Fig 2:**
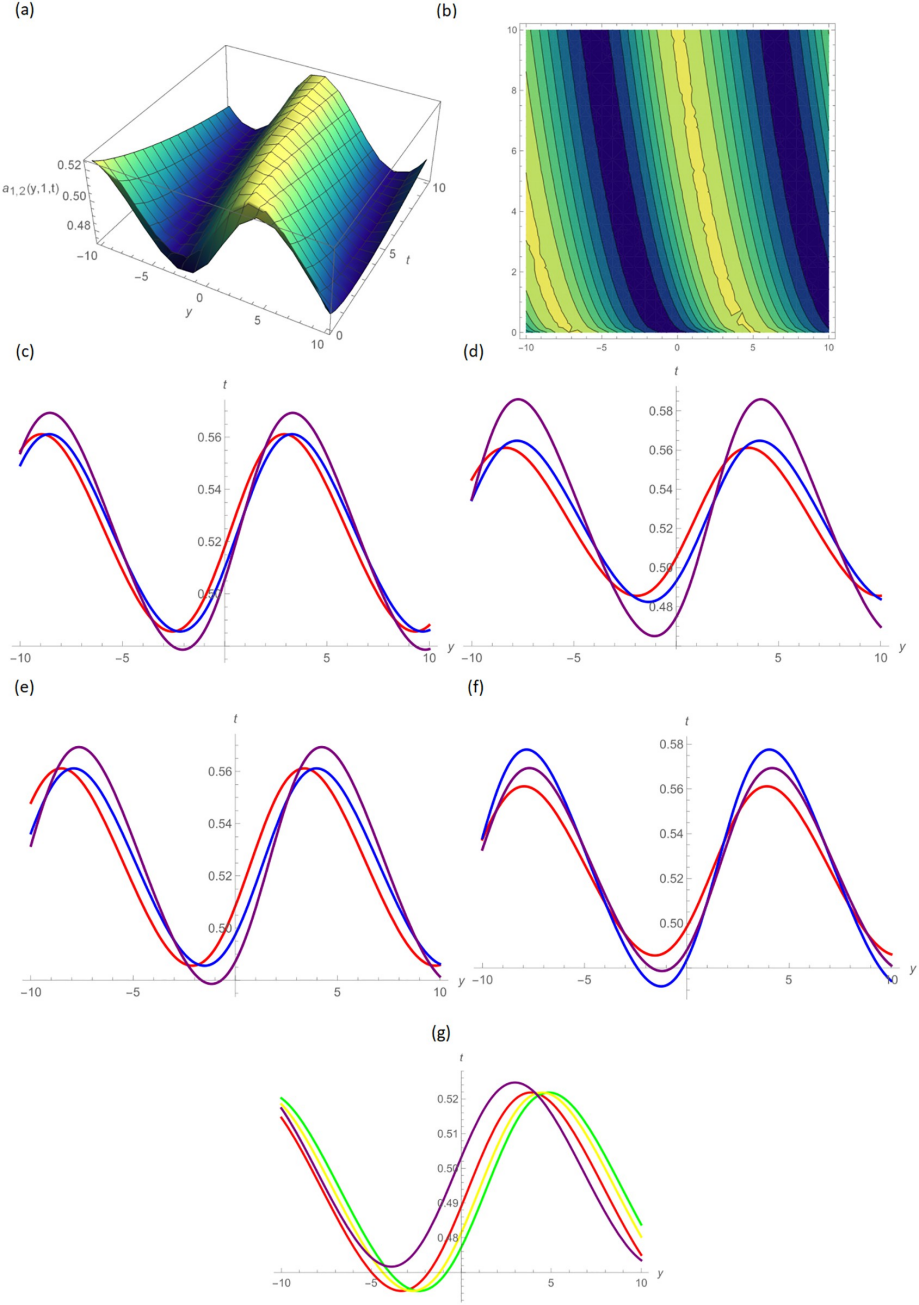
Bright wave form solution of *a*_1,2_(*y*, 1, *t*) by UM, (a) 3D plot, (b): Contour plot, when H = 0.5, R = 3.5, *ϑ* = 7, B = 0.5, d = 0.1, s = 0.09, p = 4.5, (c): BD in 2D at different values of *ς*=0.45(red),*ς*=0.65(blue),*ς*=0.9 (purple), (d): M-TD in 2D at different values of *ς*=0.5,*δ*=0.25(red),*ς*=0.75, *δ*=0.5(blue),*ς*=0.9,*δ*=0.75(purple), (e): CD in 2D at different values of *ς*=0.45,(red),*ς*=0.65(blue),*ς*=0.9 (purple), (f): L-FD in 2D at different values of *ς*=0.5(red),*ς*=0.75(blue),*ς*=0.9 (purple), (g): A comparison of BD(red), L-FD(green), M-TD(yellow) and CD(purple) at *ς* = 0.5.

**Fig 3:**
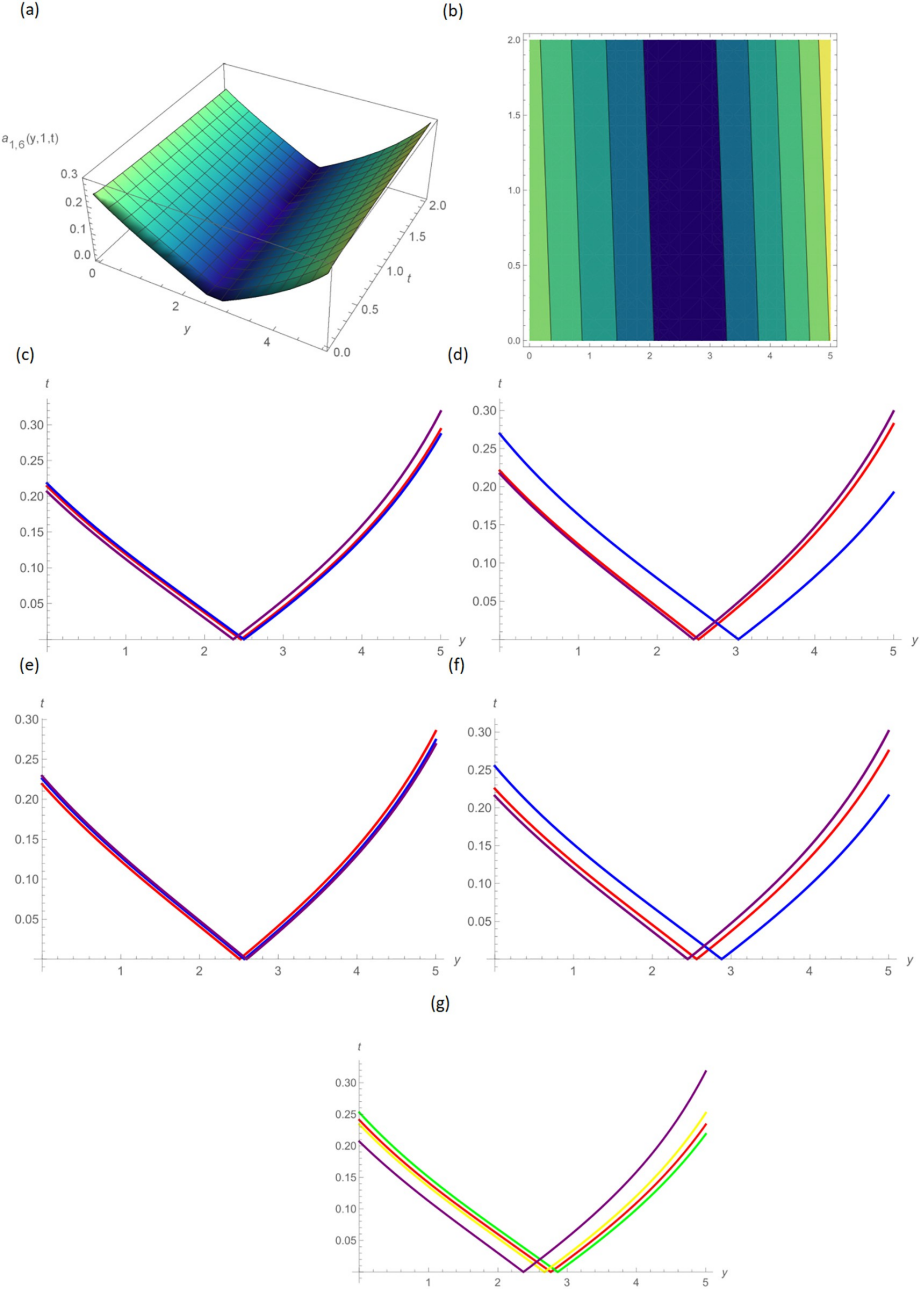
Expanded V-shaped solution of *a*_1,6_(*y*, 1, *t*) by UM, (a) 3D plot, (b): Contour plot, when H = 10.5, R = 0.5, *ϑ* = 8, B = 1.5, d = 0.1, s = 0.009, p = 0.5, (c): BD in 2D at different values of *ς*=0.45(red),*ς*=0.65(blue),*ς*=0.9 (purple), (d): M-TD in 2D at different values of *ς*=0.5,*δ*=0.25(red),*ς*=0.75, *δ*=0.5(blue),*ς*=0.9,*δ*=0.75(purple), (e): CD in 2D at different values of *ς*=0.45,(red),*ς*=0.65(blue),*ς*=0.9 (purple), (f): L-FD in 2D at different values of *ς*=0.5(red),*ς*=0.75(blue),*ς*=0.9 (purple), (g): A comparison of BD(red), L-FD(green), M-TD(yellow) and CD(purple) at *ς* = 0.5.

**Fig 4:**
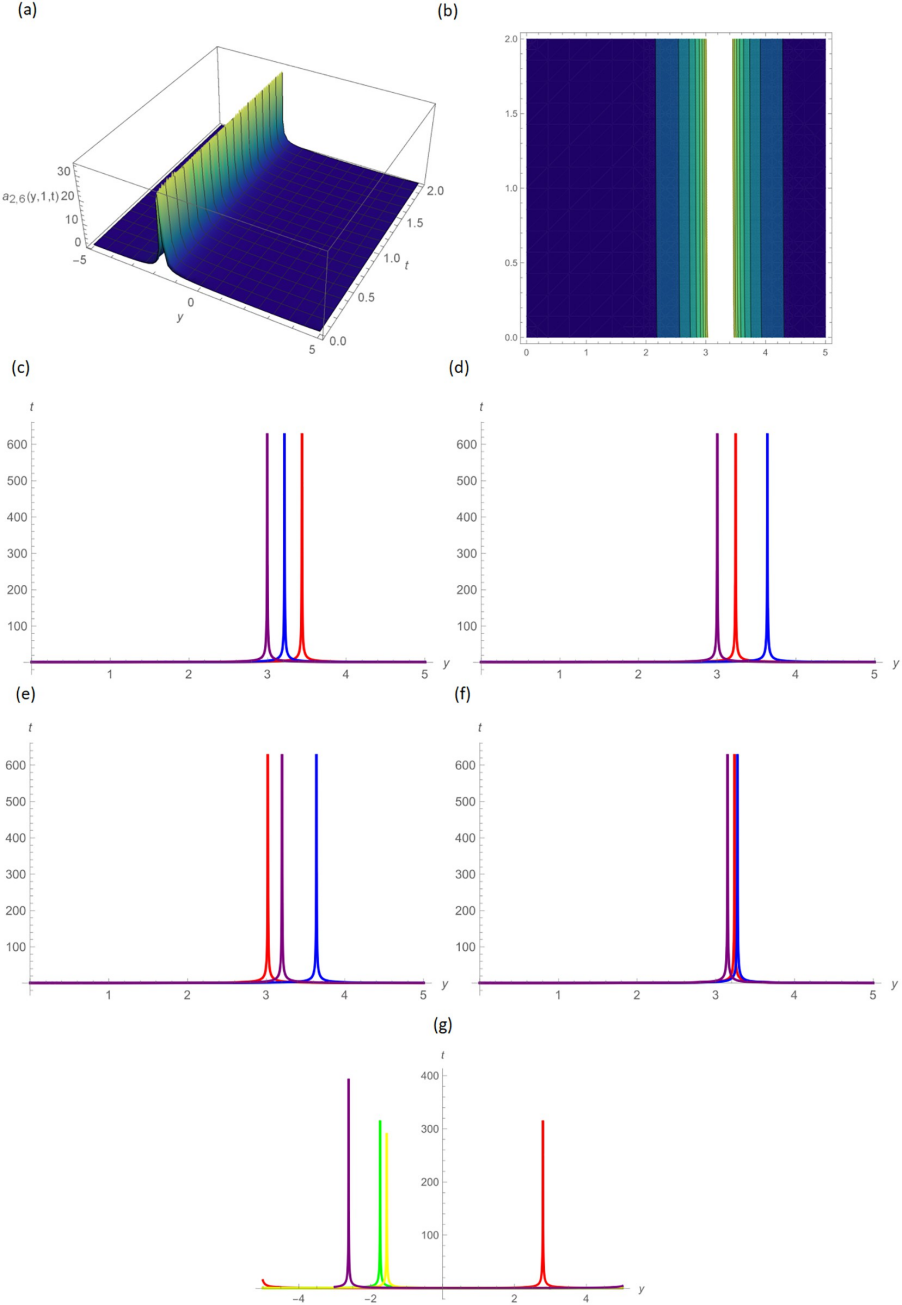
Singular wave type solution of *a*_2,6_(*y*, 1, *t*) by UM, (a) 3D plot, (b): Contour plot, when H = 10.5, R = 0.5, *ϑ* = 8, B = 1.5, d = 0.1, s = 0.009, (c): BD in 2D at different values of *ς*=0.45(red),*ς*=0.65(blue),*ς*=0.9 (purple), (d): M-TD in 2D at different values of *ς*=0.5,*δ*=0.25(red),*ς*=0.75,*δ*=0.5(blue),*ς*=0.9,*δ*=0.75(purple), (e): CD in 2D at different values of *ς*=0.45,(red),*ς*=0.65(blue),*ς*=0.9 (purple), (f): L-FD in 2D at different values of *ς*=0.5(red),*ς*=0.75(blue),*ς*=0.9 (purple), (g): A comparison of BD(red), L-FD(green), M-TD(yellow) and CD(purple) at *ς* = 0.5.

**Fig 5:**
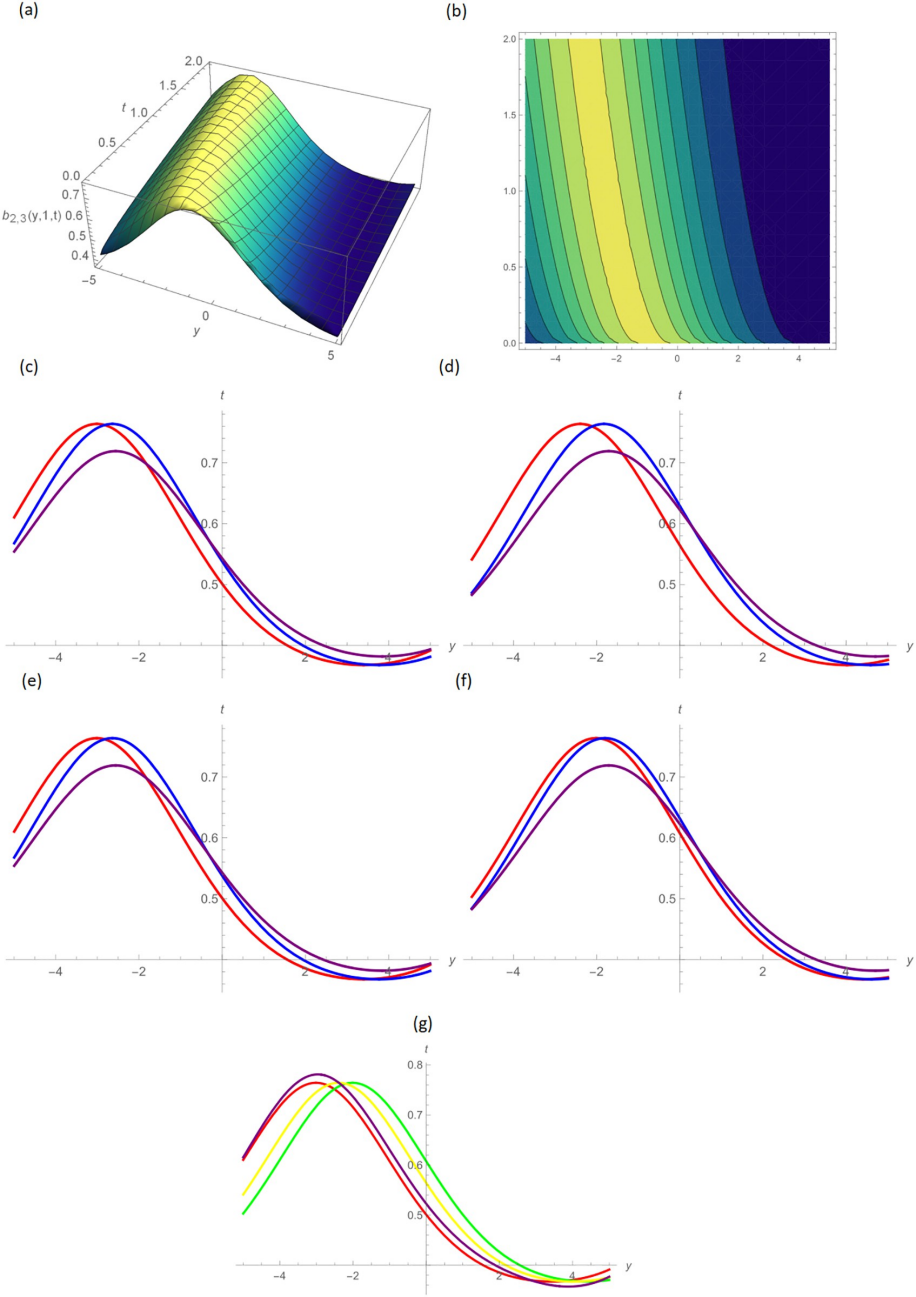
Compressed bell-shaped wave form solution of *b*_2,3_(*y*, 1, *t*) by UM, (a) 3D plot, (b): Contour plot, when H = 6.5, R = 0.7, *ϑ* = 6, B = 1.5, d = 0.1, s = 0.09, p = 0.5, (c): BD in 2D at different values of *ς*=0.45(red),*ς*=0.65(blue),*ς*=0.9 (purple), (d): M-TD in 2D at different values of *ς*=0.5,*δ*=0.25(red),*ς*=0.75, *δ*=0.5(blue),*ς*=0.9,*δ*=0.75(purple), (e): CD in 2D at different values of *ς*=0.45,(red),*ς*=0.65(blue),*ς*=0.9 (purple), (f): L-FD in 2D at different values of *ς*=0.5(red),*ς*=0.75(blue),*ς*=0.9 (purple), (g): A comparison of BD(red), L-FD(green), M-TD(yellow) and CD(purple) at *ς* = 0.5.

**Fig 6:**
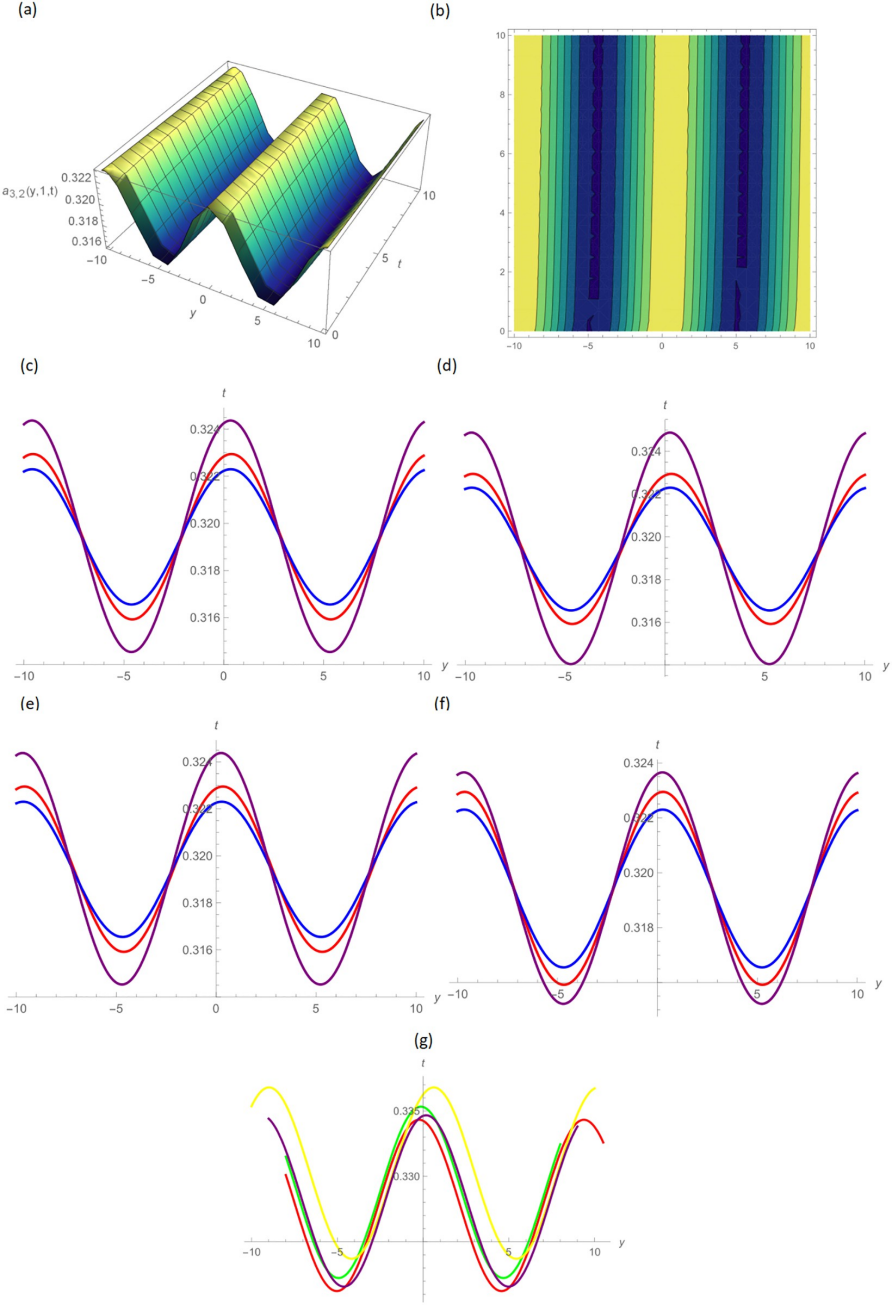
Alphabetical-shaped wave type solution of *a*_3,2_(*y*, 1, *t*) by UM,(a) 3D plot, (b): Contour plot, when H = 0.05, R = 4.5, *ϑ* = 10, B = 0.5, d = − 0.1, s = 0.009, p = − 1.5, (c): BD in 2D at different values of *ς*=0.45(red),*ς*=0.65(blue),*ς*=0.9 (purple), (d): M-TD in 2D at different values of *ς*=0.5,*δ*=0.25(red),*ς*=0.75,*δ*=0.5(blue),*ς*=0.9,*δ*=0.75(purple), (e): CD in 2D at different values of *ς*=0.45,(red),*ς*=0.65(blue),*ς*=0.9 (purple), (f): L-FD in 2D at different values of *ς*=0.45(red),*ς*=0.65(blue),*ς*=0.9 (purple), (g): A comparison of BD(red), L-FD(green), M-TD(yellow) and CD(purple) at *ς* = 0.5.

**Fig 7:**
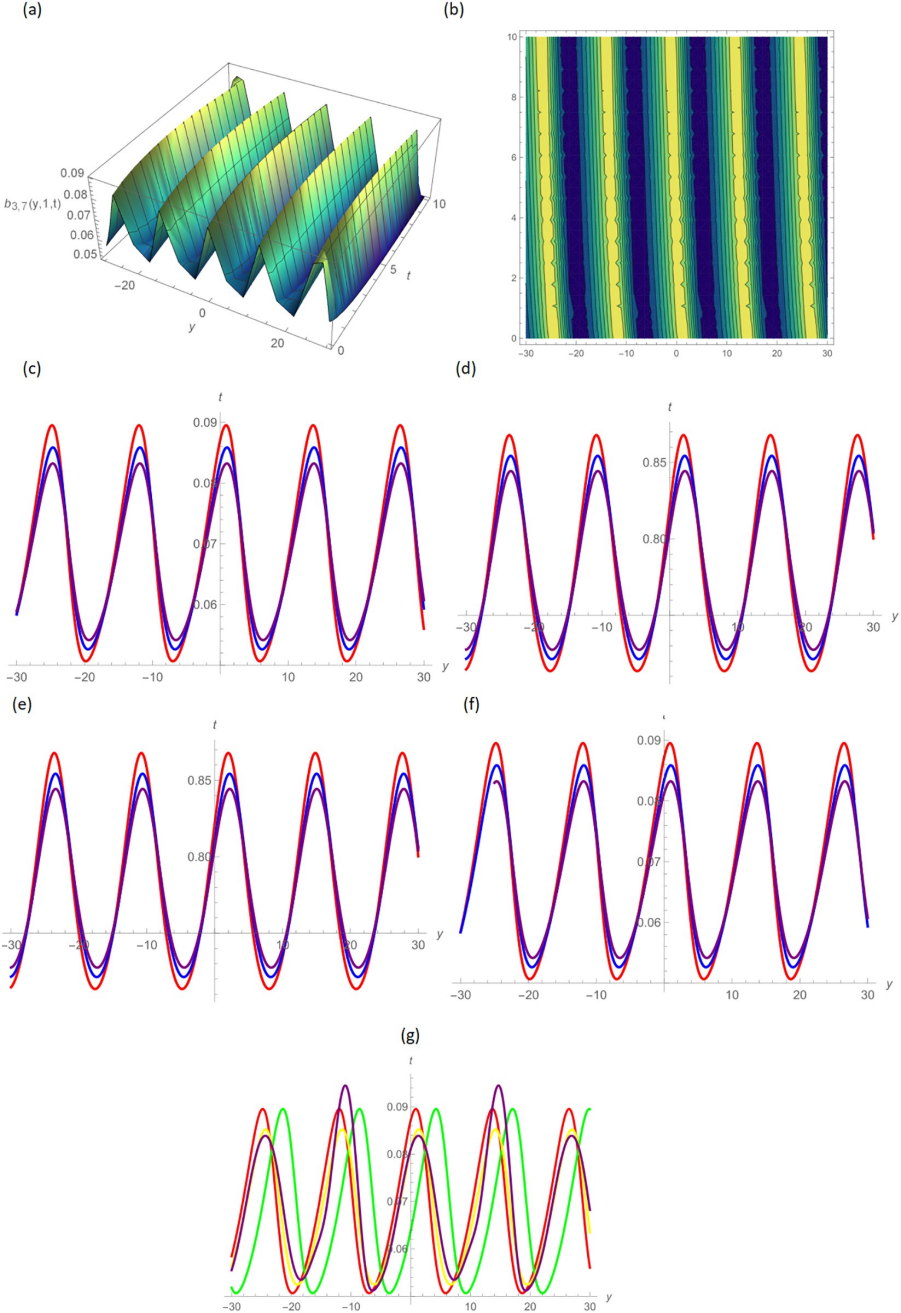
Periodic wave form solution of *b*_3,7_(*y*, 1, *t*) by UM, (a) 3D plot, (b): Contour plot, when H = 6.5, R = 0.5, *ϑ* = 6, B = 1.5, d = 0.1, s = 0.05, p = 0.05, (c): BD in 2D at different values of *ς*=0.45(red),*ς*=0.65(blue),*ς*=0.9 (purple), (d): M-TD in 2D at different values of *ς*=0.5,*δ*=0.25(red),*ς*=0.75,*δ*=0.5(blue),*ς*=0.9,*δ*=0.75(purple), (e): CD in 2D at different values of *ς*=0.45,(red),*ς*=0.65(blue),*ς*=0.9 (purple), (f): L-FD in 2D at different values of *ς*=0.5(red),*ς*=0.75(blue),*ς*=0.9(purple), (g): A comparison of BD(red), L-FD(green), M-TD(yellow) and CD(purple) at *ς* = 0.5.

In this paper, the conformable, local-fractional, beta, and M-truncated derivative operators were used to solve the nonlinear coupled BKK equation analytically. The solutions were attained by employing the effective methods known as Unified and GB sub-ODE. The exploration offers certain distinctive wave solutions. To explain the mathematical and physical implications of waves, the variety of obtained wave solutions is depicted. Solitary waves can be created using the aforementioned methods in a number of different shapes, such as a single-wave solitons see Fig 1, extended v-shaped solitons see Fig 3, singular soliton given in Fig 4, compressed bell-shaped solitons in Fig 5, W-shaped solitons in Fig 6, and periodic-shaped solitons solutions in Fig 7. Two-dimensional line graphs that compare various derivatives, such as conformable, local fractional, beta, and M-TDs, are very informative. Relating the 2D and 3D graphs of *a*_1,1_(*y*, *z*, *t*), *a*_1,2_(*y*, *z*, *t*) and *a*_1,3_(*y*, *z*, *t*), respectively, provided the single wave, bright wave form and expanded v-shaped solutions for the values *H* = 0.5, *R* = 3.5, *ϑ* = 4, *B* = 0.5, *d* = 0.1, *s* = 0.09, *p* = 1.5, within the bound −10.0 ≤ *y* ≤ 10.0, 0 ≤ *t* ≤ 10 for 3-dimensional shapes and time = 1.0 for 2-dimensional graphs, as given in Figs 1–3 demonstrated by UM.

Figs 4 and 5 provide the singular wave and compressed bell-shaped wave soliton 3D solutions of *a*_2,6_(*y*, *z*, *t*) and *b*_2,3_(*y*, *z*, *t*) for the values *H* = 10.5, *R* = 0.5, *ϑ* = 8, *B* = 1.5, *d* = 0.1, *s* = 0.009 and 2-dimensional graphs at *t* = 1.0 in the range −5 ≤ *y* ≤ 5, 0 ≤ *t* ≤ 2 by UM. Solutions for the hyperbolic and trigonometric functions in *a*_3,2_(*y*, *z*, *t*) and *b*_3,7_(*y*, *z*, *t*) being we obtain the w-shaped soliton and periodic wave solutions, respectively, by selecting the values *H* = 0.05, *R* = 4.5, *ϑ* = 10, *B* = 0.5, *d* = −0.1, *s* = 0.009, *p* = −1.5, within the bound −10.0 ≤ *y* ≤ 10.0, 0 ≤ *t* ≤ 10, and *t* = 1.0 for 2D plots in Figs 6 and 7 demonstrated by UM.

**Fig 8:**
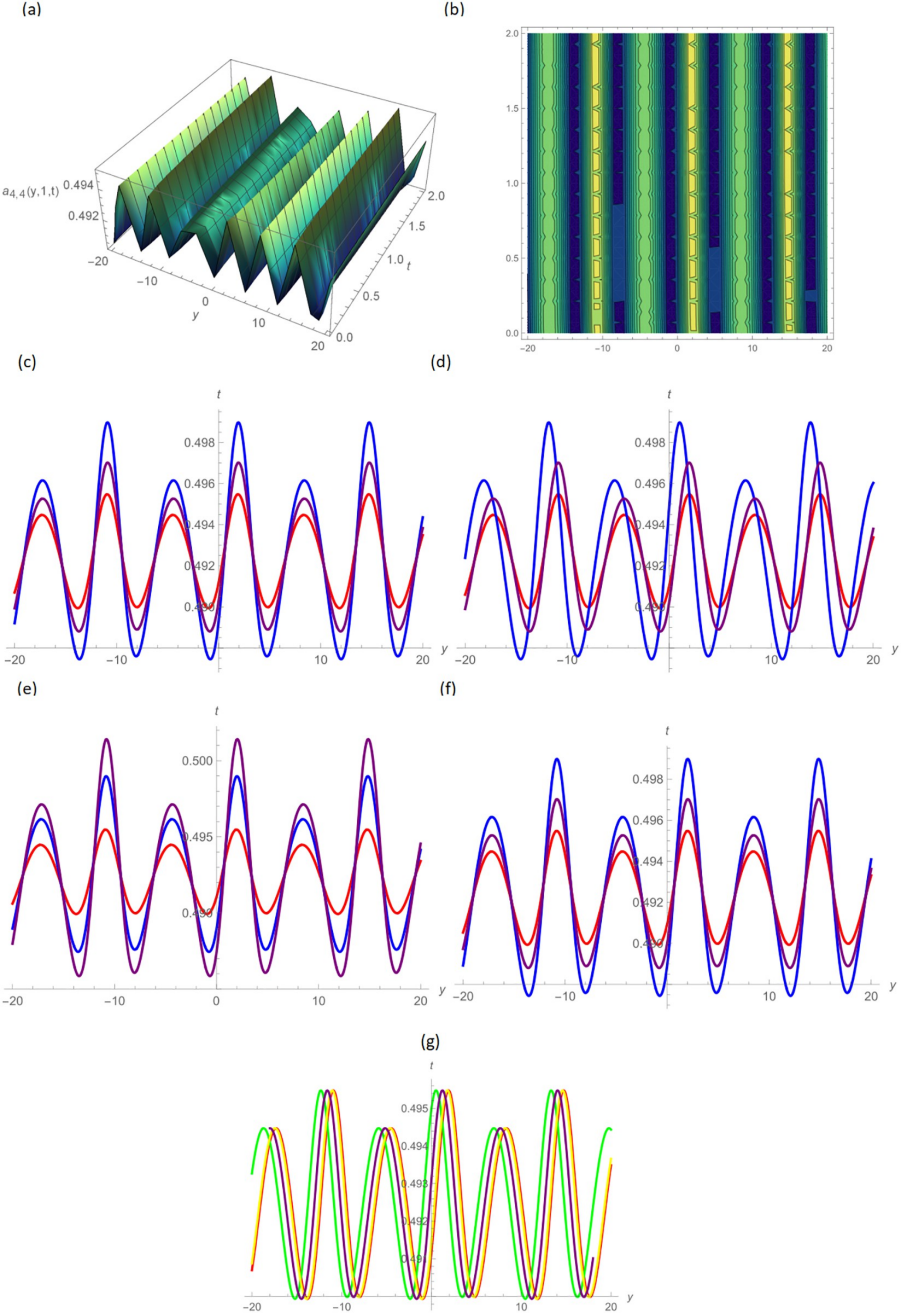
W-shaped wave form solution of *a*_4,4_(*y*, 1, *t*) by UM, (a): 3D plot, (b): Contour plot, when H = 0.05, R = 0.9, *ϑ* = 6, B = 2.5, d = 0.1, s = 0.01, p = 0.5, (c): BD in 2D at different values of *ς*=0.45(red),*ς*=0.65(blue),*ς*=0.9(purple), (d): M-TD in 2D at different values of *ς*=0.5,*δ*=0.25(red),*ς*=0.75,*δ*=0.5(blue),*ς*=0.9,*δ*=0.75(purple), (e): CD in 2D at different values of *ς*=0.45,(red),*ς*=0.65(blue),*ς*=0.9(purple), (f): L-FD in 2D at different values of *ς*=0.5(red),*ς*=0.75(blue),*ς*=0.9(purple), (g): A comparison of BD(red), L-FD(green), M-TD(yellow) and CD(purple) at *ς* = 0.5.

**Fig 9:**
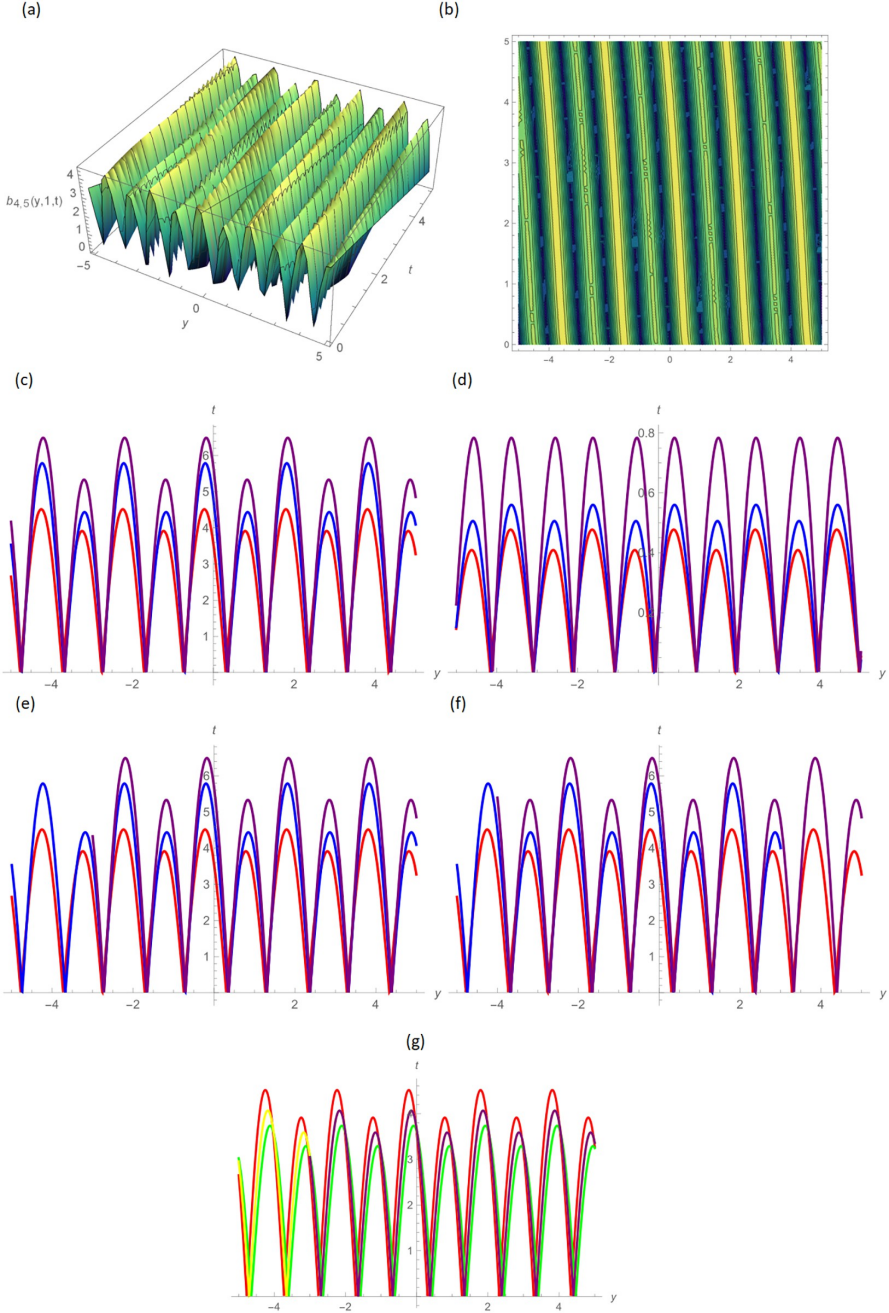
Squeezed bell-shaped periodic oscillating type solution of *b*_4,5_(*y*, 1, *t*) by UM, (a): 3D plot, (b): Contour plot, when H = 0.05,R = 0.9,*ϑ* = 6,B = 2.5,d = 0.1,s = 0.01,p = 0.5, (c): BD in 2D at different values of *ς*=0.45(red),*ς*=0.65(blue),*ς*=0.9(purple), (d): M-TD in 2D at different values of *ς*=0.5, *δ*=0.25(red), *ς*=0.75, *δ*=0.5(blue), *ς*=0.9, *δ*=0.75(purple), (e): CD in 2D at different values of *ς*=0.45(red), *ς*=0.65(blue), *ς*=0.9(purple), (f): L-FD in 2D at different values of *ς*=0.5(red), *ς*=0.75(blue), *ς*=0.9(purple), (g): A comparison of BD(red), L-FD(green), M-TD(yellow) and CD(purple) at *ς* = 0.5.

**Fig 10:**
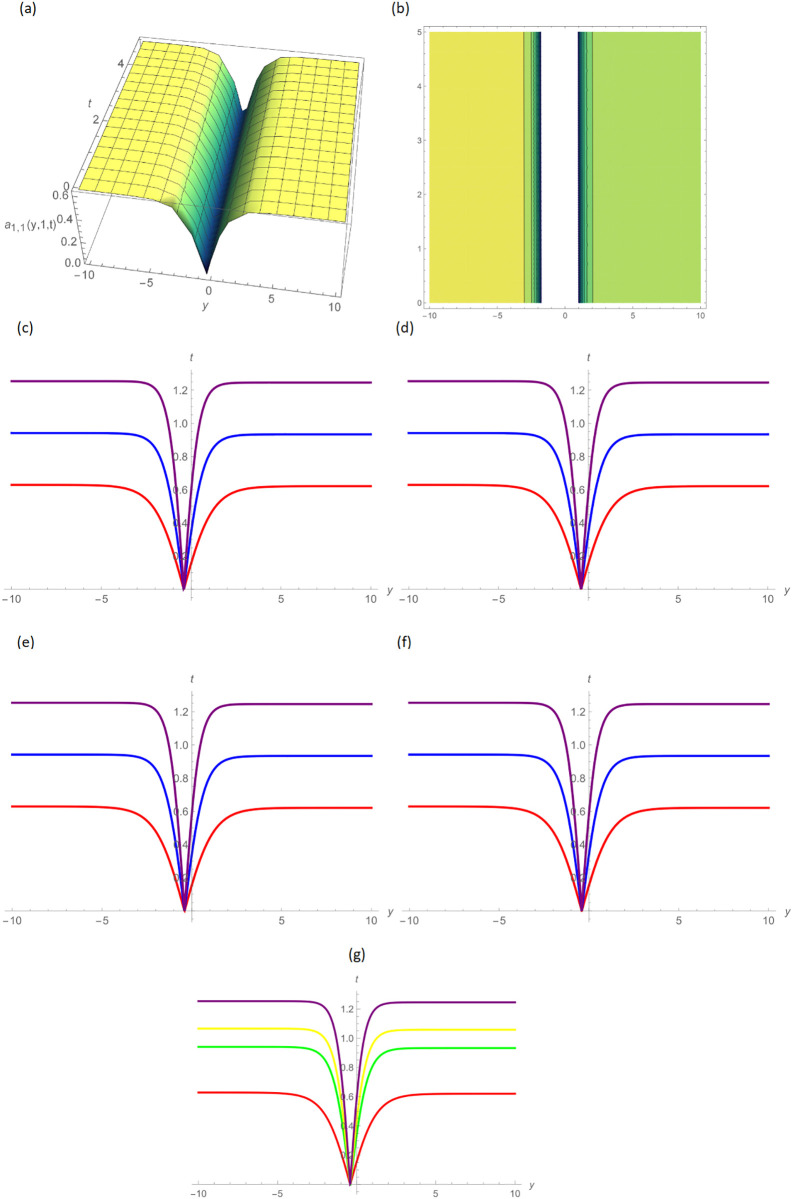
Downward bell-shaped wave solution of *a*_1,1_(*y*, 1, *t*) by GB sub-ODE method, (a) 3D plot, (b): Contour plot, when *σ* = 0.5, d = 2.5, s = 0.02, p = 1, (c): BD in 2D at different values of *ς*=0.45(red),*ς*=0.65(blue),*ς*=0.9(purple), (d): M-TD in 2D at different values of *ς*=0.5,*δ*=0.25(red),*ς*=0.75, *δ*=0.5(blue),*ς*=0.9,*δ*=0.75(purple), (e): CD in 2D at different values of *ς*=0.45,(red),*ς*=0.65(blue),*ς*=0.9(purple), (f): L-FD in 2D at different values of *ς*=0.5(red),*ς*=0.75(blue),*ς*=0.9(purple), (g): A comparison of BD(red), L-FD(green), M-TD(yellow) and CD(purple) at *ς* = 0.5.

**Fig 11:**
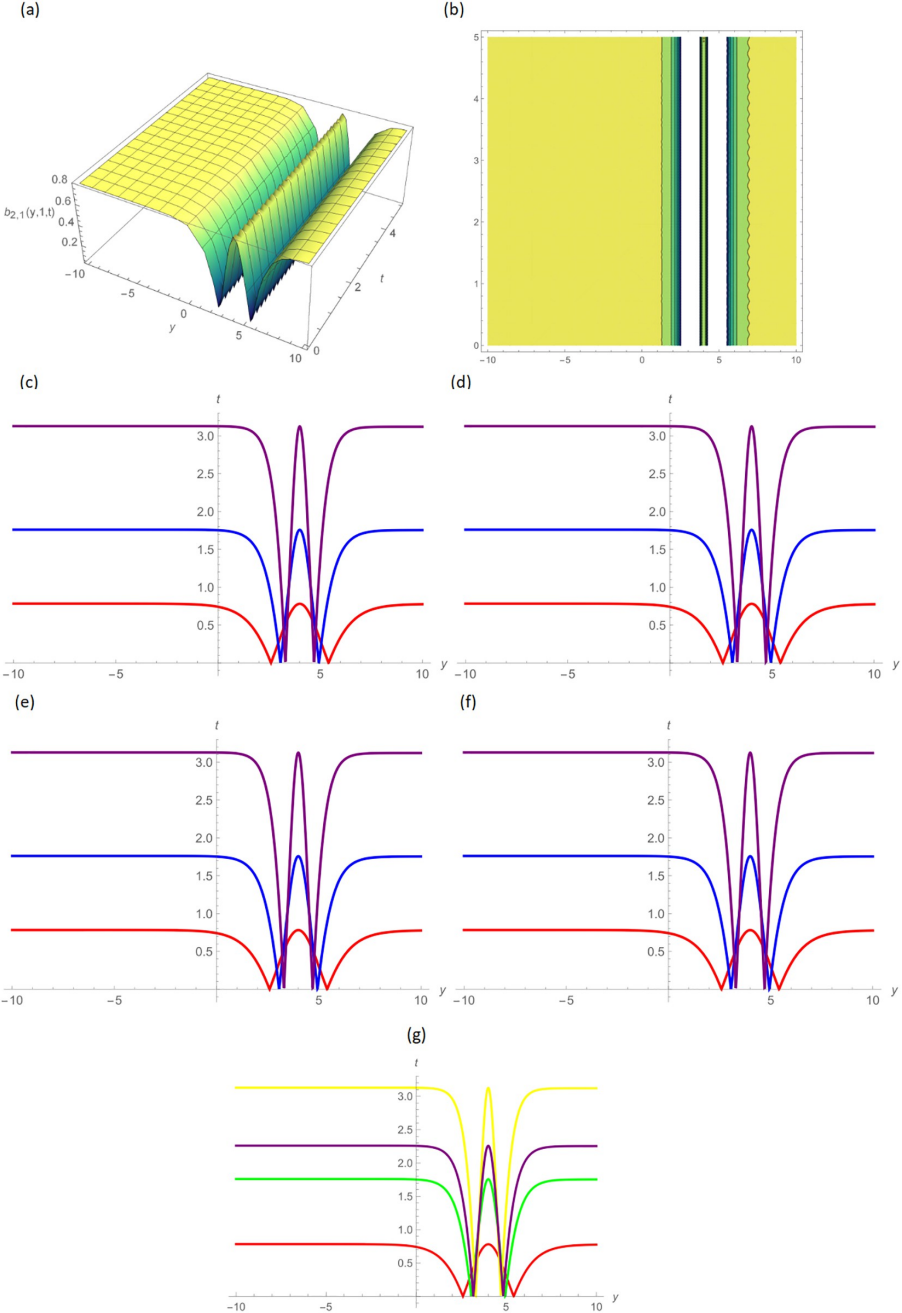
Compressed w-shaped wave solution of *b*_2,1_(*y*, 1, *t*) by GB sub-ODE method, (a): 3D plot, (b): Contour plot, when *σ*=0.5, d = − 2.5, s = 0.002, p = 10, (c): BD in 2D at different values of *ς*=0.45(red),*ς*=0.65(blue),*ς*=0.9(purple), (d): M-TD in 2D at different values of *ς*=0.5,*δ*=0.25(red),*ς*=0.75, *δ*=0.5(blue),*ς*=0.9,*δ*=0.75(purple), (e): CD in 2D at different values of *ς*=0.45,(red),*ς*=0.65(blue),*ς*=0.9(purple), (f): L-FD in 2D at different values of *ς*=0.5(red),*ς*=0.75(blue),*ς*=0.9(purple), (g): A comparison of BD(red), L-FD(green), M-TD(yellow) and CD(purple) at *ς* = 0.5.

Concerning the 3D and 2D graphs of *a*_4,4_(*y*, *z*, *t*) and *b*_4,5_(*y*, *z*, *t*), respectively, provided the alphabetical-shaped wave and squeezed bell-shaped periodic wave solutions for the parameters *H* = 0.05, *R* = 0.9, *ϑ* = 6, *B* = 2.5, *d* = 0.1, *s* = 0.01, *p* = 0.5, within the range −20.0 ≤ *y* ≤ 20.0, 0 ≤ *t* ≤ 2 for 3D shapes and *t* = 1.0 for 2D plots, as shown in Figs 8 and 9 by UM. In Figs 10 and 11 the trigonometric and hyperbolic solutions of *a*_1,1_(*y*, *z*, *t*) and *b*_2,1_(*y*, *z*, *t*), we acquire the bell-shaped and W-shaped soliton wave solutions consequently, by selecting the values *σ* = 0.5, *d* = −2.5, *s* = 0.002, *p* = 10, inside the bound −10.0 ≤ *y* ≤ 10.0, 0 ≤ *t* ≤ 5, and time = 1.0 for 2-dimensional graph by GB sub-ODE method.

## 6. Conclusions

In this article, the Unified and GB sub-ODE approaches have been used to investigate the nonlinear coupled Broer-Kaup-Kupershmidt (BKK) system, and we have confirmed some bright soliton, squeezed bell-shaped soliton, expanded v-shaped soliton, W-shaped soliton, singular soliton, and periodic solutions in terms of hyperbolic, rational, and trigonometric functions using the definitions of derivatives, i.e. beta, M-truncated, local-fractional, and conformable. In order to illustrate the compatibility of the solutions, figurative representations of some of the obtained solutions have been plotted in both two- and three-dimensional formats using independent values of the unknown parameters. We may better comprehend the dynamical properties and structures of these solutions by using the contour diagrams. In this paper, the comparison of four derivatives has been investigated. These derivatives are compared on a 2D line graph, which is quite instructive. According to the investigation, altering the quantities of fractional parameters has an impact on the soliton wave solutions; however, M-TD is regarded as more effective since a smooth wave has been seen while changing its parameter values. This function is extremely useful and effective. Better results than with other derivatives are achieved because smooth waves are produced by the Mittag-Leffler function of one parameter. The researcher may employ them in future instances as well. Future research on solving NLEEs, which have a high level of effectiveness in the nonlinear field of science and engineering, may find this work helpful in terms of approaches and precision of solutions. We can also consider BKK equation with stochastic term. In order to better understand the fluid dynamics of plasmic, optical, dispersive, and nonlinear long gravity waves, the recently discovered results, which were discovered utilising a several kinds of dynamical structures and free variables, are thought to be extremely beneficial.
